# Systematic Analysis of the *BrHAT* Gene Family and Physiological Characteristics of *Brassica rapa* L. Treated with Histone Acetylase and Deacetylase Inhibitors under Low Temperature

**DOI:** 10.3390/ijms25179200

**Published:** 2024-08-24

**Authors:** Liang Bian, Abbas Muhammad Fahim, Junyan Wu, Lijun Liu, Yuanyuan Pu, Li Ma, Yan Fang, Dan Zhang, Gang Yang, Wangtian Wang, Tingting Fan, Xiuguo Yang, Jingyu Wang, Yangyang Shi, Wancang Sun

**Affiliations:** State Key Laboratory of Arid Land Crop Science, College of Agronomy, Gansu Agricultural University, Lanzhou 730070, China; 17389333406@163.com (L.B.); fahimabbaskhan@yahoo.com (A.M.F.); wujuny@gsau.edu.cn (J.W.); puyy@gsau.edu.cn (Y.P.); mal@gsau.edu.cn (L.M.); fangy@gsau.edu.cn (Y.F.); zhangdan@gsau.edu.cn (D.Z.); yangg@gsau.edu.cn (G.Y.); wtwang@gsau.edu.cn (W.W.); fantt@gsau.edu.cn (T.F.); 18224989601@163.com (X.Y.); 13139290723@163.com (J.W.); syy409287@163.com (Y.S.)

**Keywords:** *Brassica rapa* L., *HAT* gene family, abiotic stress, expression analysis, physiological mechanism

## Abstract

*Brassica rapa* L. is an important overwintering oilseed crop in Northwest China. Histone acetyltransferases (HATs) play an important role in epigenetic regulation, as well as the regulation of plant growth, development, and responses to abiotic stresses. To clarify the role of histone acetylation in the low-temperature response of *B. rapa* L., we identified 29 *HAT* genes in *B. rapa* L. using bioinformatics tools. We also conducted a comprehensive analysis of the physicochemical properties, gene structure, chromosomal localization, conserved structural domains and motifs, *cis*-acting regulatory elements, and evolutionary relationships of these genes. Using transcriptome data, we analyzed the expression patterns of *BrHAT* family members and predicted interactions between proteins; the results indicated that *BrHATs* play an important role in the low-temperature response of *B. rapa* L. HAT inhibitor (curcumin; CUR) and histone deacetylase inhibitor (Trichostatin A; TSA) were applied to four *B. rapa* L. varieties varying in cold resistance under the same low-temperature conditions, and changes in the physiological indexes of these four varieties were analyzed. The inhibitor treatment attenuated the effect of low temperature on seed germination, and curcumin treatment was most effective, indicating that the germination period was primarily regulated by histone acetylase. Both inhibitor treatments increased the activity of protective enzymes and the content of osmoregulatory substances in plants, suggesting that histone acetylation and deacetylation play a significant role in the response of *B. rapa* L. to low-temperature stress. The qRT-PCR analyses showed that the expression patterns of BrHATs were altered under different inhibitor treatments and low-temperature stress; meanwhile, we found three significantly differentially expressed genes. In sum, the process of histone acetylation is involved in the cold response and the BrHATs gene plays a role in the cold stress response.

## 1. Introduction

Epigenetic modifications are one of the fundamental mechanisms affecting the expression and function of genes without altering the DNA sequence. These modifications include DNA methylation, histone modification, chromatin remodeling, and regulation by non-coding RNA [1]. DNA methylation is a process where cytosine methyltransferases catalyze the addition of a methyl group to a specific cytosine residue in a DNA sequence, primarily inside CpG dinucleotides [2]. This addition of a methyl group creates a surface where different protein complexes can bind and modify the histone structures, leading to changes in gene expression [3]. Eukaryotic nuclei contain DNA that is structured into nucleosomes, and the DNA is wrapped around histone proteins. Histones are a group of similar globular proteins with protruding N-terminal tails. These tails are found on the outside of the nucleosome octamer and can be chemically modified. Histones possess a wide range of post-translational modifications (PTMs) that directly control the accessibility of the transcriptional apparatus to specific genomic regions, which influences the degree to which certain loci mediate or inhibit transcription [4]. Histone modification is an important form of epigenetic regulation that plays an important role in regulating plant growth and development, gene expression regulation, and abiotic stress adaptation [5]. In eukaryotic cells, a variety of histone post-translational modifications can affect the N-terminal tails of histones, including acetylation, methylation, ubiquitination, and phosphorylation [6,7]. The reversible acetylation/deacetylation of histones can regulate the interconversion between the activated and repressed states of chromatin. The acetylation process involves the transfer of the acetyl group from acetyl-CoA to the ε-amino group of lysine residues by various enzymes such as HATs [6,7,8]. This modification is different from the N-α-acetylation of the amino terminus of proteins that occurs during translation. The acetyl group from acetyl-CoA is transferred to the primary amine at the ε position of the lysine side chain in proteins. This reversible process neutralizes the positive charge at the ε position of the lysine side chain. The loss of the positive charge and the enlarged lysine disrupt salt bridges and generate steric structures, which alters protein–protein/DNA interactions, stability, and enzymatic activity [9]. The acetylation of lysine residues also creates binding sites for other proteins, such as bromodomain-containing proteins, which bind specifically to acetylated lysine [10]. Acetylated lysines can interact with other modifications, such as competing with modifications in the same residue or interacting with modifications in neighboring residues [11,12].

Histone acetylation is co-regulated by histone acetyltransferases (HATs) and histone deacetylases (HDACs). HATs play an essential regulatory role in various growth, development, and abiotic stress-related processes by modifying the structure of chromatin through interactions with other histone modifications and transcription factors in eukaryotic cells, which affects the transcription of genes [4]. Abiotic stresses, such as drought, cold, salinity, and other environmental conditions, harm plant growth and development [13,14]. Plants have developed a variety of mechanisms to adapt to these environmental stresses, including histone modification. Plants can respond to abiotic stressors by modifying histone patterns, which in turn alters the chromatin state and gene expression [15,16]. Curcumin (Diferuloylmethane) has been validated as an acetyltransferase p300/CREB binding protein-specific inhibitor of histone/non-histone acetylation and histone acetyltransferase-dependent chromatin transcription [17,18]. Trichostatin A (TSA) was found to be a potent, specific inhibitor of histone deacetylases type I and II (HDAC class I/II) [19,20]. To date, research on histone acetyltransferases has primarily focused on model plants such as *Arabidopsis thaliana* [21] and *Zea mays* [22,23]. 

*Brassica rapa* L. is an economically important oilseed crop and the main overwintering oilseed crop in Northwest China [15,24,25,26]. An analysis of the mechanisms underlying the adaptation of winter rapeseed to abiotic stimuli is essential for enhancing its cold resistance. To date, most studies on the abiotic stress resistance of winter rapeseed have focused on the regulation of stress responses at the physiological, transcriptional, and protein levels [27,28,29]. However, few studies have examined the role of epigenetic regulation in the response to abiotic stress in winter rapeseed. Here, we identified *BrHAT* genes, characterized changes in various indexes under different inhibitor treatments, and measured the expression patterns of *BrHATs*. Overall, our findings enhance our understanding of the role of *BrHATs* in regulating the response to low-temperature stress at the physiological level.

## 2. Results

### 2.1. Genome-Wide Identification and Phylogenetic Analysis of BrHAT Genes

**Figure 1:**
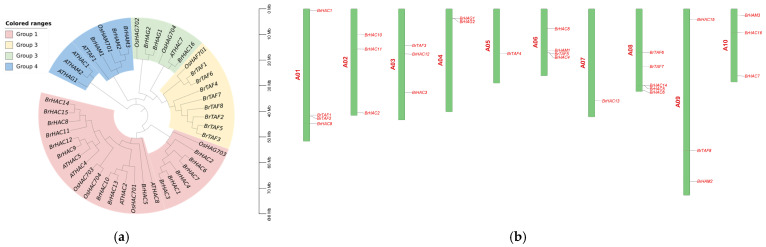
(**a**). Phylogenetic tree of HAT family members in *A. thaliana*, *O. sativa* L., and *B. rapa* L. (**b**). Chromosomal localization of *BrHAT* gene family members.

*BrHAT* genes were identified using three methods: BLAST, HMM, and CDD. A total of 29 *BrHATs* were identified, and these *BrHATs* belonged to four subfamilies: GNAT (2), MYST (3), CBP/p300 (16), and TAF (8). The lengths of the *BrHATs* sequences varied considerably, and the number of amino acids in the encoded proteins ranged from 160 to 1893, with molecular masses between 18.86 and 213.27 kDa. Theoretical pI prediction showed that *BrTAF6*, *BrHAC15*, and *BrTAF1* encode acidic proteins, and CBP/p300subfamily members encode neutral proteins, with the exception of *BrHAC15*, *BrHAC13*, *BrHAC8*, and *BrHAC5*, which encode basic proteins. The aliphatic index, which indicates the presence of fat chains, ranged from 62.03 to 102.26, suggesting that all proteins encoded by *BrHATs* are fat-soluble. Hydrophobicity prediction revealed that HAT family proteins were hydrophilic, and instability index prediction indicated that all *BrHATs* members are unstable hydrophilic proteins with values > 40 (Table 1). To explore the phylogenetic relationships among *B. rapa* L. *HAT* genes, a phylogenetic tree was constructed using the maximum likelihood method in MEGA 7.0.26 software. This analysis incorporated 46 protein sequences from three plants across two families: *A. thaliana* (9 genes), *Oryza sativa* L. (8 genes), and *B. rapa* L. (29 genes). The *HAT* genes in all three were classified into four subfamilies. The results revealed a similar branching pattern between *A. thaliana* and *B. rapa* L. *HAT* genes in the Cruciferae family were compared with those in rice, and unequal numbers of branches at all levels and of genes in each branch were observed in the subfamilies. The CBP/p300 subfamily had more branches than the other subfamilies, which indicated that CBP/p300 subfamily members were more structurally diverse (Figure 1a). This unequal branching and differentiation may stem from the natural selection experienced by the species during the evolutionary process, suggesting that each subfamily has some short specific sequences with the same homologous structural domains, which may lay the structural foundation for the functional diversity of the *BrHATs* family. 

Figure 1b illustrates the chromosomal distribution of the 29 *B. rapa* L. *HAT* genes. These genes span 10 chromosomes, each with one to four members of *BrHAT* genes. The distribution of *BrHAT* genes across chromosomes is independent of chromosome length. For example, chromosomes A05 and A07 had only one *BrHAT* gene; chromosome A04 had two *BrHAT* genes; chromosomes A02, A03, A09, and A10 had three *BrHAT* genes, chromosomes A01 and A06 had four *BrHAT* genes; and chromosome A08 had the highest number of *BrHAT* genes (five), suggesting that tandem duplications played an important role in the expansion of this gene family.

### 2.2. Conserved Domains, Structure, and Replicate Sequences of BrHAT Genes

**Figure 2:**
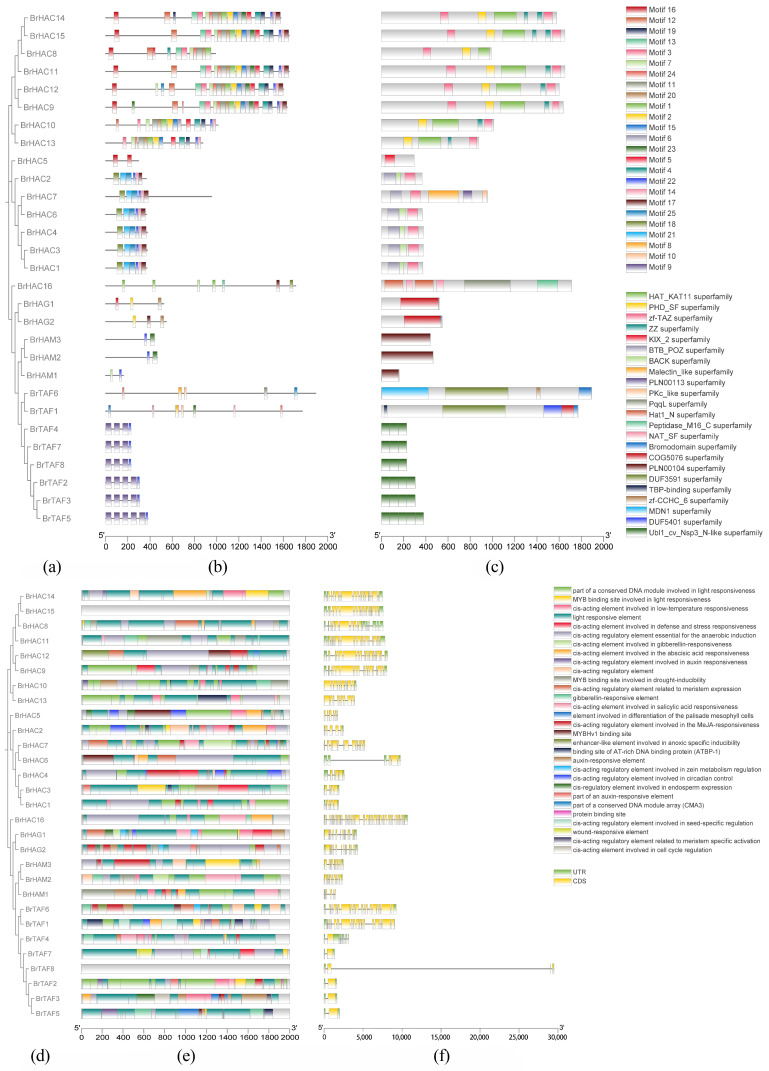
Gene structure analysis. (**a**). Phylogenetic tree of *HAT* family members in *B. rapa* L. (**b**). Conserved motif distribution of *BrHATs*. (**c**). Functional domain analysis of *BrHATs*. (**d**). Phylogenetic tree of *BrHATs* family members in *B. rapa* L. (**e**). Promoter *cis*-acting element analysis of *BrHAT* genes (**f**). Gene structure map of *BrHATs*.

To clarify the structural characteristics of the proteins encoded by *BrHAT* genes, we analyzed their conserved structural domains, and the results are shown in Figure 2. The numbers and types of conserved structures and motifs varied among subfamilies, and the sequence lengths of the conserved motifs also differed. Almost all subfamily members, with the exception of GNAT subfamily members, contain motif 22, indicating that this part of the structure was relatively conserved and plays an important role in the function of *BrHATs*. Only members of the TAF subfamily contain motif 9; only members of the MYST subfamily contain motif 23; and the MYST, TAF, and GNAT subfamilies have a relatively small number of motifs (Figure 2b), suggesting that the structural features of these genes have evolutionarily diverged. 

The conserved structural domains of the CBP/p300 subfamily were more diverse than those of the other subfamilies, and they may have a variety of functions (Figure 2c). The functional prediction of conserved structural domains revealed that the CBP/p300 subfamily contained the HAT-KAT 11 and zf-TAZ structural domains, and none of the other three subfamilies contained these domains. HAT-KAT 11 is required for H3K56 acetylation [30]. The zf-TAZ have important roles in protein recognition and protein–protein interactions [31]. Different types of zinc fingers may bind to different proteins or histones and be recruited to different target areas of chromatin. The GNAT subfamily contained the KIX-2 domain, which was not found in the other subfamilies. The kinase-inducible domain interacting (KIX) domain is a highly conserved independently folding three-helix bundle that serves as a docking site for transcription factors, whereupon promoter activation and target specificity are achieved during gene regulation [32]. MYST subfamily was unique to the PLN00104 structural domain, tentatively designated as MYST-like histone acetyltransferase [33]. TAF family was unique to the UBl1-cv-NSP3-n-like domains. This ubiquitin-like (Ubl) domain (Ubl1) is found at the N-terminus of coronavirus Nsp3, a large multi-functional multi-domain protein which is an essential component of the replication/transcription complex (RTC) [30]. The different conserved structural domains suggested that members of different subfamilies may perform various functions. An analysis of promoter *cis*-acting elements revealed the inclusion of a growth hormone response element; a binding site for a DNA-binding protein (ATBP-1); the MYB-binding sites involved in the light response and drought induction; and the gibberellin, low-temperature, abscisic acid, anaerobic-induced, auxin, and salicylic acid responses (Figure 2e).

To delve deeper into the evolutionary characteristics of the *BrHATs* gene family, the structures of *BrHAT* genes were examined. The arrangement and number of introns and exons significantly affect gene family evolution and can provide insights into phylogenetic relationships [34,35]. According to the findings presented in Figure 2f, there was considerable variability in the number and structure of introns and exons across individual genes. For example, *BrTAF6* and *BrHAC14* have more than 10 exons but only two introns and *BrHAC6* has only two exons and three introns. This variation suggests that *BrHATs* have distinct functional roles [36].

### 2.3. Analysis of Covariance within and between Species in BrHAT Genes

**Figure 3:**
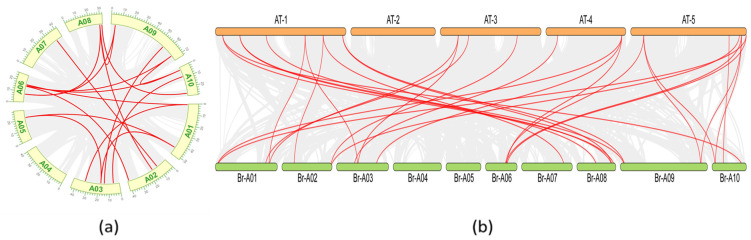
Replication relationships among *BrHATs* gene family fragments. (**a**). Analysis of *BrHATs* gene intraspecific replication events. (**b**). *BrHAT* genes and *A. thaliana* genomic duplication events.

Gene colinearity is an indicator of gene duplication events [37]. A total of 17 pairs of colinear *BrHAT* genes were identified (Figure 3a). The covariance of *BrHAT* genes was observed on all chromosomes, with the exception of chromosome A04. Tandemly duplicated genes accounted for 72.4% of gene families, suggesting that gene-associated duplication is a possible origin for some *BrHATs* and an important mechanism for gene family expansion. To further explore the replication events of *BrHATs*, genomic covariance between *B. rapa* L. and *A. thaliana* (Figure 3b) was analyzed. The gray bands connect all genes with covariance in *B. rapa* L., and the red lines link *HAT* genes exhibiting covariance, indicating that highly homologous replicative relationships were likely generated by the same gene duplication event. genes lacking covariance are presumed to have mutated. Intraspecific covariance analysis revealed tandem duplications on neighboring proximal and distal chromosomes. A comparison with the *A. thaliana* genome revealed 29 covariance relationships between *BrHAT* genes and *AtHATs* genes, indicating that the number of *BrHATs* was increased in *B. rapa* L. 

### 2.4. Expression Analysis of BrHATs under Abiotic Stress

**Figure 4:**
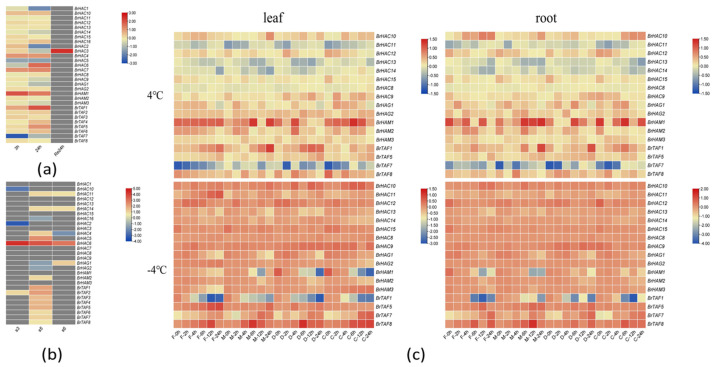
Expression analysis of *BrHATs* family members. (**a**). Transcriptome data of *BrHATs* family members in winter rapeseed (Longyou-7) at 4 °C. (**b**) Transcriptome data of *BrHATs* family members in winter rapeseed (Longyou-7) at different overwintering stages; sampling date: S3 (24 November 2019), S5 (4 January 2020), and S6 (25 April 2020). (**c**). Expression analysis of *BrHATs* family members in leaves and roots under low-temperature stress. The gray rectangles in Figure 4a,b indicate that no significant differential expression of this gene occurred under this treatment compared to the control. “C” is “CT-2360,” “M” is “MXW-1,” “D” is” DT-9,” and “F” is “2018-FJT.”.

Transcriptome data provide insights into gene expression dynamics. We explored the expression patterns of members of the *BrHATs* family using laboratory transcriptome data from *B. rapa* L. (Longyou-7) under low temperature stress at 4 °C and at different overwintering stages. We found that the expression of *BrHAT* genes varied with growth. The expression of only *BrHAC3* increased after 24 h of growth. No significant changes in the expression of the other genes compared with the control were observed (i.e., in the absence of low-temperature treatment at 4 °C). The expression of *BrHAC1*, *BrHAC14*, *BrHAC2*, *BrHAC5*, *BrHAG1*, *BrHAM3*, and *BrTAF7* decreased following low-temperature treatment; however, the magnitude of this decrease varied depending on the treatment duration. The expression of most genes was consistently down-regulated as the treatment time extended. However, the expression of *BrTAF7*, *BrHAC15*, *BrHAC2*, *BrHAC9*, *BrHAG1*, and *BrHAM3* was up-regulated and then down-regulated as the duration of low-temperature treatment extended. The remaining genes exhibited varying degrees of up-regulation after different durations of treatment at 4 °C (Figure 4a). Furthermore, an analysis of the transcriptome data at different overwintering periods revealed that only some members of the CBP/p300 subfamily (*BrHAC2*, *BrHAC6*, and *BrHAC10*) and some members of the TAF subfamily (*BrTAF2*) were differentially expressed at S3. Specifically, the expression of *BrHAC6* and *BrTAF2* was up-regulated; the expression of *BrHAC6* was up-regulated to a higher degree, and its expression remained high during S5 and S6. During S5, the expression of all the TAF subfamily members, the five CBP/p300subfamily members (*BrHAC4*, *BrHAC5*, *BrHAC6*, *BrHAC11*, and *BrHAC14*), and one gene (*BrHAM2*) of the MYAT subfamily was up-regulated, and the expression of all GNAT subfamily members, *BrHAC2*, *BrHAC10*, and *BrHAC15*, was down-regulated; the rest of the family members were differentially expressed. During S6, the expression of three CBP/p300 subfamily members (*BrHAC6*, *BrHAC11*, and *BrHAC14*) and one GNAT subfamily member (*BrHAG1*) was up-regulated, and the expression of one CBP/p300 subfamily member (*BrHAC4*) was down-regulated (Figure 4b). These findings suggest that the responses of *BrHAT* genes in Longyou-7 to different low-temperature treatments and different overwintering stages vary. These results suggest that *BrHATs* are involved in regulating the low-temperature response of *B. rapa* L. The expression of *BrHAT* genes was determined using qRT-PCR, and Ct values were analyzed using the 2^−ΔΔCt^ method [38]. The expression of some *BrHATs* was assessed following various temperature treatments; in Figure 4c, red indicates a higher expression, and blue indicates a lower expression. Under the same temperature conditions, the expression of *BrHATs* was slightly lower in the roots than in the leaves, but the difference was not statistically significant. Under both 4 °C and 0 °C (Figure S1) treatments, the overall gene expression patterns were similar. The expression of CBP/p300 subfamily members was low. However, the expression of *BrHAC8*, *BrHAC9*, *BrHAC11*, and *BrHAC12* in the highly cold-tolerant variety 2018-FJT increased with the duration of treatment. The expression of *BrHATs* in the highly cold-resistant variety 2018-FJT also increased with the duration of treatment (Figure 4c). The expression levels of *BrHAC8*, *BrHAC9*, *BrHAC11*, and *BrHAC12* remained relatively stable over time in the cold-resistant variety 2018-FJT. However, in the weakly cold-tolerant varieties, the expression of *BrHAC11* initially decreased and then gradually recovered, and the expression of *BrHAC12* initially increased and then decreased. As the duration of the low-temperature treatment extended, the expression of all subfamily members, with the exception of *BrTAF7*, became up-regulated. The expression of *BrTAF1* was up-regulated in cold-resistant varieties compared with weakly cold-resistant varieties. At the onset of the 4 °C treatments, the expression of the four subfamily members was up-regulated in the highly cold-resistant varieties compared with the weakly cold-resistant varieties. The number of down-regulated genes in the four varieties was nearly equal after 24 h of 4 °C treatments. After −4 °C treatment, the expression of most of these genes was up-regulated, with the exception of some treatments involving *BrHAM1*, *BrTAF1*, and *BrTAF7*. The expression of each gene varied among varieties, and the expression of these genes was altered as the duration of the low-temperature treatment extended. When the treatment time reached 24 h, the expression of *BrHAC11* and *BrTAF5* was significantly higher in the CT-2360 than in the other three highly cold-tolerant varieties. The expression of *BrTAF7*, *BrTAF8*, and *BrHAC9* decreased in the 4 °C treatments, but the expression of these genes increased steadily as the duration of low-temperature treatment at −4 °C increased. Furthermore, the expression of these genes was higher in 2018−FJT than in the other three weakly cold-tolerant varieties. The genes that showed significant changes in expression under the two temperature treatments were *BrHAM1*, *BrTAF1*, and *BrTAF7*. The expression of *BrHAM1* and *BrTAF1* was up-regulated to a greater extent under the 4 °C treatment. Additionally, the expression of *BrHAM1* and *BrTAF1* increased in weakly cold-resistant varieties following prolonged exposure to the −4 °C treatment. The expression of these genes decreased as the duration of exposure to low temperatures increased. In contrast, the expression of *BrTAF7* substantially decreased following exposure to 4 °C, and its expression increased as the duration of exposure to −4 °C increased. The differences in the expression patterns of these genes after different low-temperature treatments suggest that they play a role in the response to low-temperature stress in *B. rapa* L.

### 2.5. Gene Ontology (GO) Enrichment Analysis of BrHAT Genes

**Figure 5:**
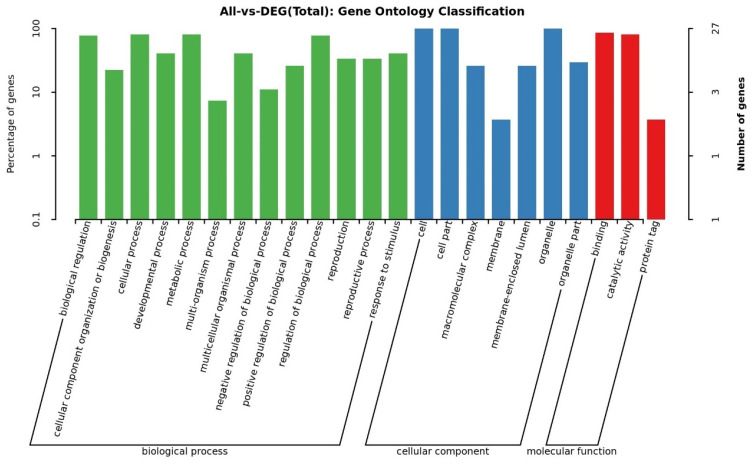
GO enrichment analyses of *BrHATs*; larger −log_10_(*p*-value) values indicate more significant pathways.

GO annotations of protein sequences provide insights into the biological functions of *BrHAT* genes. As shown in Figure 5. *BrHATs* in the category of biological processes include GO terms such as biological regulation, cellular processes, developmental processes, metabolic processes, multi biological processes, multi cellular biological processes, biological process regulation, reproductive processes, and response to stimuli. *BrHATs* were enriched in the following GO terms in the molecular function category: the regulation of binding, catalytic activity, and protein tag. Enriched GO terms in the cellular component category included the cell part, the macromolecular complex, the membrane, and the organelle part. These findings highlight the key role of *BrHATs* in regulating cell activities and catalytic processes. 

### 2.6. Protein Interaction Prediction of BrHAT Genes

**Figure 6:**
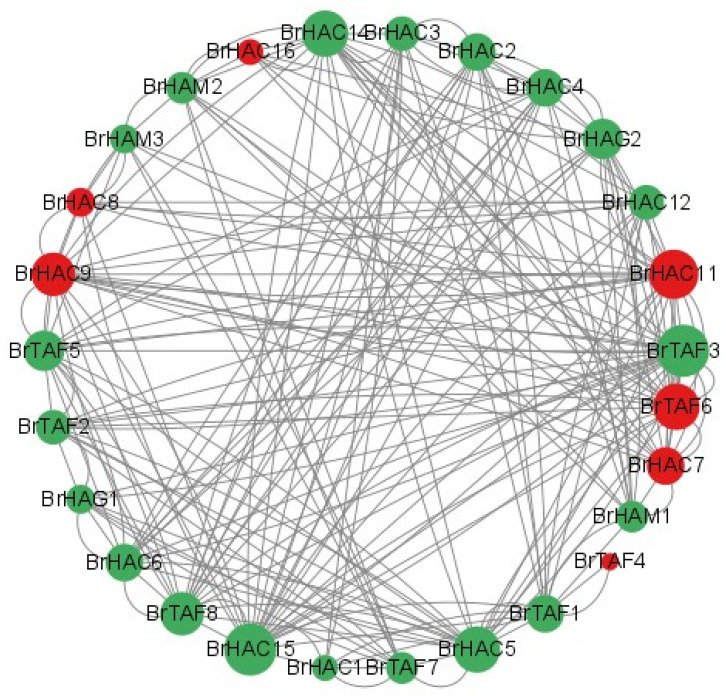
Protein interaction prediction results. Positive correlations are in red, and negative correlations are in green; the number of nodes is correlated with the size of the circle of the graph where the gene is located.

To further clarify the role of *BrHATs* in *B. rapa* L., protein interactions were predicted for *BrHATs*. A total of 27 significant differentially expressed genes (*p* < 0.05) were screened using transcriptome data, and their co-expression patterns were analyzed; these genes were found to encode proteins that engaged in abundant interactions. The expression of seven *BrHATs* was positively correlated with the expression of the other members; the expression patterns of the rest of the genes were negatively correlated (Figure 6). Greater numbers of interactions were predicted for *BrHAC11*, *BrHAC15*, and *BrTAF3*, and the genes with few predicted interactions were *BrHAC8*, *BrHAC15*, and *BrTAF4*.

### 2.7. Effects of Different Inhibitor Treatments on HAT and HDAC Activities in B. rapa L.

**Figure 7:**
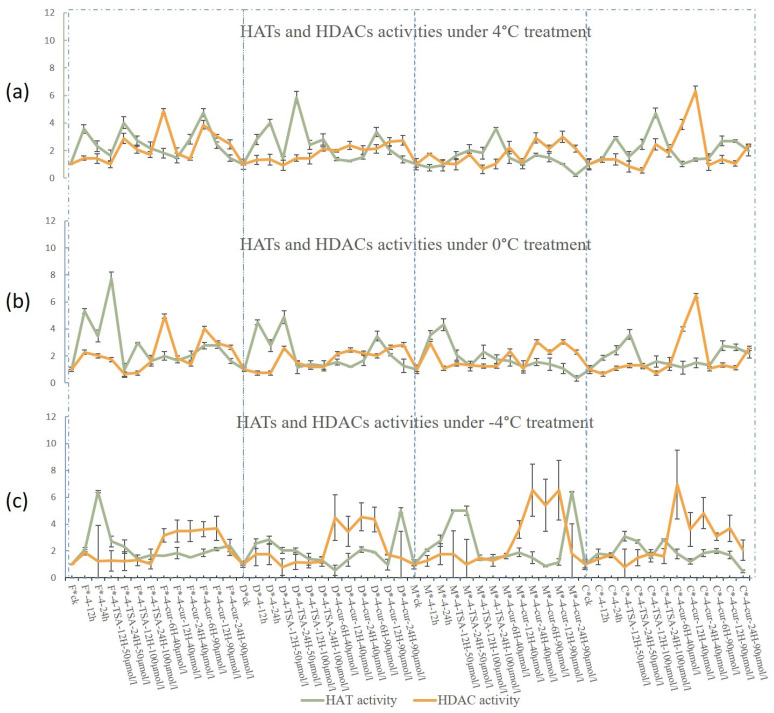
The effects of different inhibitors on HAT and HDAC activities. (**a**). HAT and HDAC activities under 4 °C treatment. (**b**). HAT and HDAC activities under 0 °C treatment. (**c**). HAT and HDAC activities under −4 °C treatment. “C” is “CT-2360,” “M” is “MXW-1,” “D” is” DT-9,” and “F” is “2018-FJT”. “*” Connect the variety name and processing name in the caption.

We examined the activities of HATs and HDACs in four materials after different treatments using an ELISA kit. The activities of HATs were higher in each variety following low-temperature stress treatment than in the CK, and the magnitude of the increase in HAT activity was enhanced in cold-resistant varieties. The results indicate that the activity of HATs was directly related to cold resistance under 4 °C, 0 °C, and −4 °C, whereas the activity of HDACs remained constant. These findings indicate that HATs play a key role in the response of *B. rapa* L. to low-temperature stress. An analysis of the effect of inhibitor treatments on HAT and HDAC activities revealed a dynamic equilibrium, with distinct variation in the effects of the two inhibitors on the four materials during the 4 °C treatment. The cold-tolerant varieties were more susceptible to the effects of the inhibitors, and the results under −4 °C treatment were distinct from those at 4 °C (Figure 7). There was no significant difference in the activities of these two enzymes after 4 °C treatment and 0 °C treatment when the rest of the conditions were the same. Moreover, the weakly cold-tolerant variety exhibited higher HAT and HDAC activities compared with the highly cold-tolerant variety 2018-FJT. This suggests that HATs and HDACs play a role in the response to low-temperature treatment.

### 2.8. Effects of Inhibitor Treatments on the Seed Germination and Growth of Different Cold-Resistant Varieties

**Figure 8:**
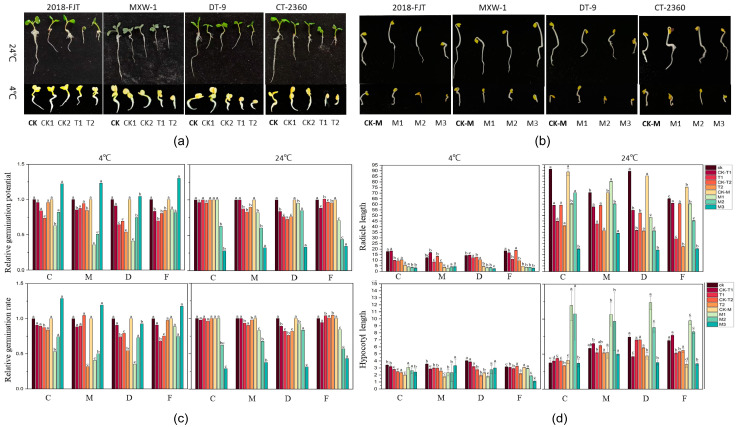
Effects of different inhibitors on the seed germination characteristics of different varieties. (**a**). Germination of each material after TSA treatment. (**b**). Germination of each material after curcumin treatment. (**c**). GE and GP of each material after different treatments. (**d**). Length of the radicle and hypocotyl in each material after different treatments. “C” is “CT-2360,” “M” is “MXW-1,” “D” is ”DT-9,” and “F” is “2018-FJT.” CK: 1/2 MS medium, T1: 1/2 MS medium containing 1 μM TSA, CK1: control with DMSO equal to T1, T2: 1/2 MS medium containing 3 μM TSA, CK2: control with DMSO equal to T2. CK-M: ddH_2_O immersion for 24 h, M1: 50 μM curcumin immersion for 24 h, M2: 200 μM curcumin immersion for 24 h, and M3: 350 μM curcumin immersion for 24 h; seeds were germinated, with three replicates per variety, at 4 °C and 24 °C. Different normal letters indicate significant differences within different treatments for the same variety (*p* < 0.05).

Germination tests were conducted by treating plants with different concentrations of inhibitors. Minimal differences in GE were observed in the highly cold-tolerant variety, 2018-FJT, at 24 °C and 4 °C; however, the GE and germination rate of the weakly cold-tolerant variety CT-2360 were significantly lower in the 4 °C treatment than in the 24 °C treatment (Figure 8). The GE was higher in T1 and T2 than in their respective controls (CK1 and CK2) under all TSA treatments for all three materials, with the exception of CT-2360, which exhibited the opposite pattern. At a temperature of 4 °C, the GE of CT-2360 was higher in T1 than in CK1, but it was lower in T1 than in CK. Similar patterns were observed in the GP. Under 4 °C, the GE and GP of all the varieties were higher in T1 than in CK1 under different TSA concentrations, with the exception of CT-2360, which exhibited the opposite pattern.

The curcumin-soaking treatment was shaded to prevent light from affecting the action of curcumin, and the growth and development of each material were delayed during germination; the germination process was also delayed in all varieties (Figure 8). At 24 °C, the germination rate and GP of all varieties markedly decreased as the concentration of curcumin applied increased. Conversely, at 4 °C, the concentration of curcumin increased, and the germination rate and appearance of all varieties were enhanced to varying degrees. Furthermore, the germination rate and GP of all varieties were higher in the M3 treatment than in the curcumin control (CK-M) at different temperatures, with the exception of DT-9. In general, as the level of curcumin immersion increased, the germination rate and characteristics of less cold-tolerant varieties improved to a greater degree compared with the more resistant varieties. The lengths of the hypocotyl and radicle in all four varieties dramatically decreased following the 4 °C treatments compared with the 24 °C treatments (Figure 8a,c). After TSA treatment, the lengths of both the hypocotyl and radicle were significantly lower (*p* < 0.05) at 4 °C than in the control groups (CK1 and CK2), and the T2 concentration had the most pronounced effect. At 24 °C, the rate of change in the hypocotyl was less significant, and the rate of change in root length was more pronounced (Figure 8b,c). At 24 °C, the root length of each variety decreased as the concentration of curcumin applied increased. However, after 4 °C treatment, the root length of MXW-1 increased as the concentration of curcumin applied increased. The responses of the root length of the other three varieties to the 24 °C and 4 °C treatments were similar. However, there was substantial variation between weak and strong cold-resistant cultivars at 24 °C. The hypocotyl length of the varieties first increased and then decreased as the concentration of curcumin applied increased. Conversely, the hypocotyl length of the highly cold-resistant varieties progressively shortened as the curcumin concentration applied increased in the 4 °C treatment; variable trends were observed in the other three varieties. These findings suggest that both TSA and curcumin can partially mitigate the effects of low temperatures, and curcumin has a more pronounced effect (Figure 8d). The results support our hypothesis that HATs play a more significant role in regulating seed germination.

### 2.9. Effect of Exogenous Inhibitors on Antioxidant Enzyme Activities in B. rapa L.

**Figure 9:**
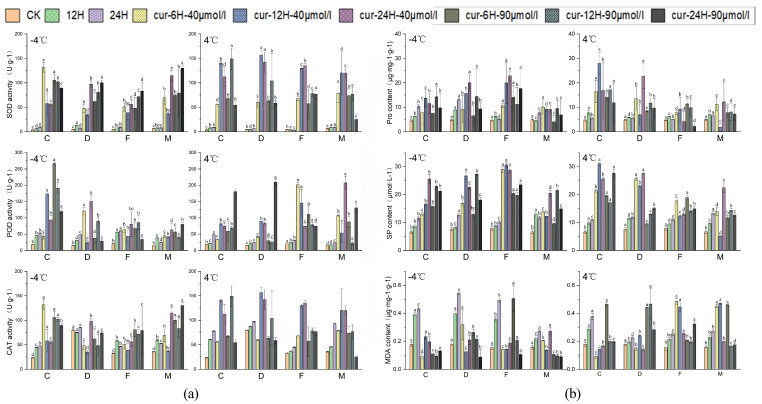
Effect of different concentrations of curcumin on the physiological indexes of *B. rapa* L. (**a**). Antioxidant enzyme activities under treatments with different concentrations of curcumin. (**b**). The soluble matter content under treatments with different concentrations of curcumin. “C” is “CT-2360,” “M” is “MXW-1,” “D” is” DT-9,” and “F” is “2018-FJT.” Different normal letters indicate significant differences within different treatments for the same variety (*p* < 0.05).

Under low-temperature stress conditions, the energy balance, metabolite homeostasis, and redox homeostasis are altered via the activation of a series of molecular mechanisms, cold-resistance-related protective enzymes, and metabolite activities to improve the ability of plants to tolerate low temperatures [39]. Therefore, we determined the activities of three antioxidant enzymes (CAT, SOD, and POD) to analyze the physiological effects of two inhibitors on *B. rapa* L. differing in cold tolerance after low-temperature stress. After 12 h of treatment, the enzyme activities of all varieties were significantly elevated. The application of 40 μM curcumin spray had the most pronounced effect on the activities of SOD and CAT. An inverse correlation between treatment duration and enzyme activity was identified, and this might be attributed to the degradation of curcumin under light. There were noticeable differences in CAT activity among varieties. A significant increase in CAT activity was observed after 12 h of treatment with 40 μM curcumin in the DT-9 variety, which has high cold resistance. The increase in CAT activity was significantly higher in the CT-2360 variety, which has weak cold tolerance, than in other varieties after treatment with 90 μM curcumin. Patterns of SOD activity were similar to patterns of CAT activity, but the activity of POD modestly deviated from the patterns of SOD and CAT activity. The POD activity of the 2018-FJT and MXW-1 varieties was highest at 6 h of curcumin treatment. This high level of POD activity was maintained in 2018-FJT, even after 24 h of treatment, and the POD activity of MXW-1 was highest at this time point. POD activity was markedly higher in CT-2360 and DT-9 than in other varieties following 24 h of treatment with 90 μM curcumin. Curcumin treatment enhanced the activity of antioxidant enzymes in all varieties at a temperature of 4 °C. The CAT activity of CT-2360 was highest after 12 h of treatment with various dosages of curcumin, especially at a concentration of 90 μM. However, CAT activity after 12 h treatment was notably higher in this variety than in the other three varieties. Additionally, changes in SOD activity differed from changes in CAT activity, and these patterns remained consistent at a concentration of 40 μM curcumin. At a concentration of 40 μM curcumin, the SOD activity of the three cold−tolerant varieties was highest after 24 h of treatment. However, the SOD activity of CT-2360 was highest after 6 h; it then decreased markedly at 12 h before stabilizing. After treatment, the POD activity was much higher in all four varieties under −4 °C than under control conditions. The increase in POD activity in weakly cold-resistant cultivars was particularly significant. When exposed to 40 μM curcumin treatment, the POD activity of the three cold-resistant varieties was highest after 24 h of treatment. However, the peak in POD activity in CT-2360 was observed after only 12 h. When treated with 90 μM curcumin, POD activity peaked in the two strongly cold-tolerant varieties after 12 h, whereas the peak in POD activity occurred after 6 h in the weakly cold-tolerant varieties. The results of the study showed that exogenous curcumin spraying increased the antioxidant enzyme activities of winter rapeseed varieties under different low-temperature treatments. The effect of curcumin treatment on antioxidant enzyme activities varied among cold-resistant varieties, and its mitigating effect was stronger on weakly cold-resistant varieties than on strongly cold-resistant varieties under low-temperature stress (Figure 9a). Furthermore, the activity of each antioxidant enzyme was enhanced by the exogenous administration of TSA (Figure S2). However, curcumin treatment had a more pronounced effect on each indicator than the TSA treatment. Our results demonstrate that the increase in antioxidant enzyme activity was particularly evident at different curcumin concentrations and was also more pronounced at 12 h after curcumin treatment. 

### 2.10. Effect of Exogenous Curcumin Application on the Soluble Matter Content of B. rapa L.

Under low-temperature conditions, plants regulate the content of soluble substances and the concentration of MDA through the activation of a series of molecular mechanisms related to cold resistance, thereby regulating cellular osmotic pressure, reducing the degree of membrane lipid peroxidation, and improving their low-temperature tolerance [39]. Under −4 °C treatment, the MDA content of each variety was lower after curcumin treatment compared with the CK (Figure 9b). The reduction in the MDA content was more pronounced in the 90 μM curcumin treatment than in the 40 μM curcumin treatment following −4 °C treatment. Similarly, the content of proline and soluble protein markedly increased following curcumin treatment. After exposure to −4 °C, CT-2360, which is weakly cold-resistant, had higher levels of proline and soluble protein after 12 h of treatment. The content of proline peaked at 24 h in weakly cold-resistant varieties, and the soluble protein content varied substantially after curcumin treatment. Patterns of variation in the TSA treatment were similar to patterns of variation in the curcumin treatment, suggesting that both TSA and curcumin treatments can reduce low-temperature stress-induced cell damage by decreasing membrane lipid peroxidation, regulating osmotic pressure in the plant cytoplasm, and modulating the cellular redox potential. Furthermore, the results of the TSA treatment were comparable to those of the curcumin treatment, indicating that both treatments can effectively decrease cell damage caused by low-temperature stress by reducing the oxidation of membrane lipids, regulating the osmotic pressure within the plant cytoplasm, and adjusting the cellular redox potential (Figure S3). The results indicated that the extent of the reduction depends on the cold tolerance of the treated materials and the concentration of the inhibitor.

### 2.11. Expression Analysis of BrHATs after Inhibitor Treatment

**Figure 10:**
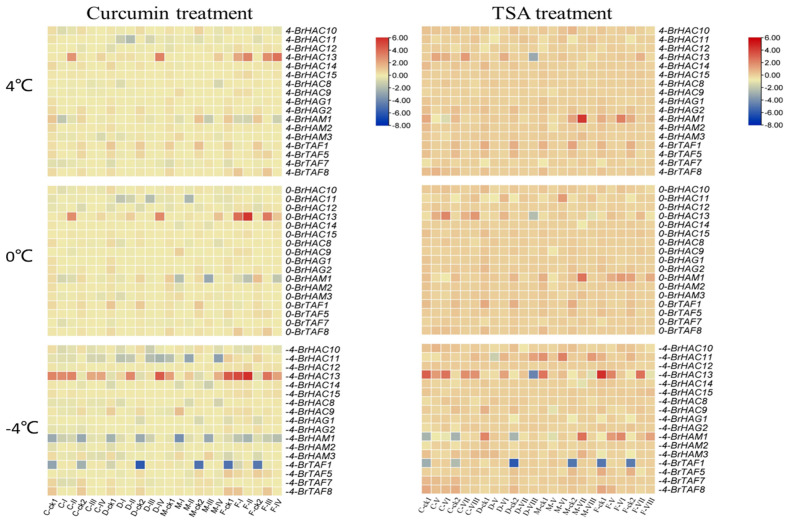
Expression analysis of *BrHATs* in leaves after inhibitor treatment. “C” is “CT-2360”, “M” is “MXW-1”, “D” is “DT-9”, and “F” is “2018-FJT”. ck1: 12 h; ck2: 24 h; I: 40 µmol/L curcumin spray treatment for 12 h; II: 40 µmol/L curcumin spray treatment for 24 h; III: 90 µmol/L curcumin spray treatment for 12 h; IV: 90 µmol/L curcumin spray treatment for 24 h; V: 50 µmol/L TSA spray treatment for 12 h; VI: 50 µmol/L TSA spray treatment for 24 h; VII: 100 µmol/L TSA spray treatment for 12 h; VIII: 100 µmol/L TSA spray treatment for 24 h.

qRT-PCR results showed that the expression of each gene under curcumin and TSA treatments exhibited contrasting trends. Curcumin inhibited the expression of *HAT* genes, which resulted in the down-regulation of most *BrHATs* to varying degrees after treatment. The expression patterns of each gene were generally consistent across different temperatures, and the most significant changes were observed at −4 °C (Figure 10). The expression levels of *BrHAC13* and *BrTAF1* increased after the application of curcumin in different varieties. In the absence of curcumin treatment, the expression of *BrHAC13* was down-regulated in all four varieties after 24 h. However, after curcumin treatment, the expression of *BrHAC13* was up-regulated in all varieties, and it was up-regulated to a greater degree in varieties with high cold tolerance. The expression of *BrTAF1* was also similar in the 12 h and 24 h treatment. The expression patterns of *BrHAG1* and *BrHAC12* were distinct from those of the other genes, and marked differences were observed among varieties. The expression of these two genes was increased in the cold-resistant variety 2018-FJT after the application of the curcumin spray. Furthermore, aside from *BrHAC13*, *BrTAF1*, *BrHAG1*, and *BrHAC12*, the expression of all other *BrHATs* decreased following the curcumin spray treatment. The functional effects of TSA were opposite to those of curcumin. Following TSA treatment, the expression of all *BrHATs* increased to varying degrees. However, the expression of certain genes, such as *BrHAC12* and *BrHAC15*, did not significantly differ compared with the unsprayed treatment in the presence of TSA. The genes showing a significantly altered expression following TSA spray treatment were comparable to those showing a significantly altered expression following curcumin treatment. Specifically, the expression patterns of *BrHAC13*, *BrHAM1*, and *BrTAF1* were similar in the TSA and curcumin treatments. However, the expression pattern of *BrHAC13* in the TSA treatment was opposite to that observed in the curcumin treatment. The expression patterns of the other two genes were similar in the curcumin and TSA treatment, but the up-regulation of these genes was more pronounced; consequently, the differences in the expression of these genes between these treatments and the control group were marked. The expression patterns of *BrHATs* were distinct, and specific expression patterns were observed following various inhibitor treatments; these expression patterns were also in contrast to those observed in the untreated control. This indicates that histone acetylases play a role in the responses of plants to low-temperature stress and are regulated by *BrHAT* genes. The results of this experiment indicate that *BrHAC13, BrHAM1*, and *BrTAF1* play important roles in the acetylation of *B. rapa* L.

## 3. Discussion

### 3.1. Analysis of HAT Genes in B. rapa L.

Plants are exposed to various environmental factors during their life cycle, which induce changes in gene expression. One crucial mechanism mediating these changes is the post-translational modification of histones, specifically histone acetylation, which comprises an epigenetic regulatory network that mediates responses to environmental stress [40]. Histone acetylation plays a role in temperature regulation during plant growth and development. Several genes involved in histone acetylation have been discovered in rice, maize, and *A. thaliana*. These genes control the activation of cold stress-induced genes and affect the cold acclimation process [41,42,43,44,45]. HATs, which are essential enzymes involved in histone acetylation, are involved in physiological processes, such as transcriptional activation, cell cycle regulation, and DNA replication. HATs comprise four gene families: GNAT, MYST, CBP, and TAFII250 [15]. By examining the sequences of known HATs in *A. thaliana*, a total of 29 *BrHATs* were discovered in the genome of the winter rapeseed cultivar Longyou-7. An analysis of the number of genes and phylogenetic analysis revealed that *B. rapa* L. has significantly more *BrHATs* than *A. thaliana*, suggesting that the regulation of growth, development, and stress responses differs in these two species. The substantial variation in motif number between subfamilies indicates that these subfamilies have undergone distinct evolutionary paths. Subcellular localization prediction results in *B. rapa* were consistent with those in *A. thaliana*; proteins were localized to the nucleus, implying that *BrHATs* may share functional similarities with *AtHATs*. *AtGCN5* has been shown to interact with stress-response-related transcription factors, such as *CBF1*, *DREB2A*, and *AREB1*-2, which encode proteins that comprise a complex that catalyzes the acetylation modification of H3K9, H3K14, H3K27, H4K4, and H4K5 loci [46]. Moreover, genes such as *AtHAC1* and *AtHAF2* have been implicated in responses to various types of stress, such as salt stress, low-temperature stress, and light stress [47]. An analysis of the functional domains specific to the four subfamilies revealed that the CBP/p300 subfamily contains domains essential for H3K56 acetylation and important in protein recognition and protein–protein interactions. The GNAT and TAF subfamilies contain domains that play important roles in transcription [30,31]. An analysis of the functional structural domains and promoter *cis*-acting elements of *BrHATs* revealed that they are equally involved in these stress response processes. However, additional studies are needed to determine whether *BrHATs* are involved in responses to other types of stress, such as salt and drought stress. GO enrichment analyses of *BrHATs* showed that they are involved in a variety of biological processes, including stress responses, and molecular functions, such as intracellular binding regulation, catalytic activity, and protein labeling. The results of protein interaction prediction analysis revealed that the members involved in interactions were not consistent; negative correlations were more common than positive correlations. All of these findings suggest that *BrHATs* play different roles in regulating various physiological and biochemical processes. 

### 3.2. Relationships between HAT and HDAC Activities and Cold Tolerance

Acetylation is the most common epigenetic post-translational modification. The acetylation level is regulated by the dynamic balance between enzymatic acetylation and deacetylation via HATs and HDACs [48]. Histone acetyltransferases are important enzymes that play a major role in physiological processes such as transcriptional activation, cell cycle regulation, DNA replication, and repair [49]. Previous studies have shown that HDACs play a role in regulating the response of apples to low-temperature stress, thereby enhancing cold tolerance [50]. Curcumin (diferuloylmethane) and Trichostatin A (TSA) are inhibitors of histone acetylase and histone deacetylase [17,18,19,20]. In our study, both TSA and curcumin treatments had positive effects on the cold resistance of *B. rapa*, which alleviated the effects of low temperature by regulating histone acetylation and deacetylation. However, the effects varied depending on the cold-resistant materials and the action of the two inhibitors. In strongly cold-resistant varieties, antioxidant enzyme activity significantly increased after treatment with both inhibitors, and the MDA content substantially decreased, which ultimately improved cold resistance. Additionally, the significant increase in the proline content observed in the weakly cold-resistant variety after treatment indicates that both inhibitors enhanced the cold resistance of *B. rapa* L.

### 3.3. Effects of TSA Treatment on the Growth, Development, and Physiological Indexes of B. rapa *L.*

Previous studies have demonstrated that TSA treatment significantly increases the acetylation levels of histones H3 and H4 [51]. In our study, we observed that root length growth was inhibited in all four varieties following TSA treatment. This finding is consistent with the results of previous studies, indicating that TSA treatment inhibits the growth of the primary roots of *A. thaliana* [51]. Although some studies have shown that *A. thaliana* seeds germinate faster after TSA treatment [52], our findings indicate that TSA treatment generally hinders the germination rate of *B. rapa* L. seeds. However, the germination rate of some varieties increased under low-temperature treatment. The results of our germination tests indicated that the application of TSA suppressed the root length in all four *B. rapa* L. varieties. However, certain treatments led to an enhancement in seed germination rates. The inhibitory effects of TSA treatment on root length were stronger than the low-temperature treatment, indicating that TSA enhances histone acetylation and partially mitigates the effect of low temperature on seed germination and growth. When plants detect low temperatures and other forms of stress, they produce significant amounts of protective chemicals to control physiological and biochemical changes. This helps them maintain a balance in cellular osmotic potential and stability in cell membranes [53]. Previous studies have revealed a strong positive relationship between the ability to withstand cold temperatures and the increase in SOD, POD, and CAT enzyme activities, as well as the content of soluble protein and free proline [54]. Varying trends in the indices of different varieties after TSA treatment under low temperatures were associated with their ability to withstand cold, indicating that alterations in histone acetylation levels induced by TSA may control the expression and function of cold-resistant genes, ultimately improving the cold resistance of *B. rapa* L. and ensuring normal growth.

### 3.4. Effects of Curcumin Treatment on the Growth, Development, and Physiological Indexes of B. rapa *L.*

At high concentrations, curcumin inhibits cell growth and is utilized as a clinical drug and plant growth regulator [55]. At low concentrations, it regulates plant growth and development by enhancing the activity of internal protective enzymes and increasing the content of osmoregulatory substances, which improves the resistance of crops to environmental stress, quality, and yield [56,57,58,59,60,61,62,63,64]. Numerous studies have investigated the effects of curcumin on various plants [40,41,59,60,61,63,64,65]. In our study, the growth of all four varieties decreased, and the growth of the roots was inhibited during germination at 24 °C under curcumin treatment. Conversely, the root length of two cold-resistant varieties increased at higher curcumin concentrations at 4 °C, and root inhibition was stronger at 24 °C than at 4 °C. Additionally, the relative germination rate of each variety at 24 °C was negatively correlated with the curcumin treatment concentration, and the relative germination rate of curcumin-treated seeds at 4 °C increased as the curcumin concentration applied increased. The germination test results demonstrated that curcumin alleviated the effect of low temperature on seed germination. The germination rate of seeds from each variety increased as the curcumin concentration increased. The hypocotyl length of the highly cold-resistant varieties was significantly shorter than that of the other three varieties at high curcumin concentrations. Curcumin more effectively alleviated the effects of low temperature than TSA, suggesting that HATs primarily regulate the seed germination process. The results of spraying treatments showed that exogenous curcumin had a similar spraying effect as TSA at different low temperatures and both increased the antioxidant enzyme activities of the plants to a certain extent, which may be related to the fact that both curcumin and TSA have antioxidant activities [66,67]. Exogenous curcumin application at various low temperatures also induced different increases in antioxidant enzyme activities and osmoregulatory substances in cold-resistant winter rapeseed varieties. Differences were also observed among varieties, and the mitigating effect of low-temperature stress was stronger on weakly cold-resistant varieties than on strongly cold-resistant varieties. Consistent with the results of the germination test, more variability was observed in the curcumin-treated group.

### 3.5. The Role of BrHATs in Growth, Development, and the Response to Low-Temperature Stress

Based on the effects of various inhibitor treatments on HAT and HDAC activities in *B. rapa* L., we hypothesized that HATs and HDACs play a role in responses to low-temperature treatment. An analysis of transcriptome data under low-temperature stress revealed the significant up- and down-regulation of *HAT* genes, with the exception of *BrHAC11*, suggesting that most *BrHATs* are involved in the low-temperature stress response. A transcriptome analysis of data from different overwintering periods revealed significant changes in the expression patterns of *BrHATs*, indicating that they play a role in regulating responses to various abiotic factors during these periods.

The expression of *BrHATs* changed after low-temperature stress, and differences in *BrHATs* expression in the roots and leaves were observed. In most cases, the expression of each *BrHATs* was higher in the leaves than in the roots, suggesting that they are involved in the response to low temperature. The spray treatment of leaves revealed that the two inhibitors affected changes in physiological indexes; the expression of members of this gene family was also affected. Therefore, *BrHATs* were involved in regulating stress responses in winter rapeseed and the response to low temperature in *B. rapa* L. The results of this study revealed the function of *BrHATs* and their regulatory mechanism and will aid future studies.

## 4. Materials and Methods

### 4.1. HAT Gene Identification and Analysis of Phylogenetic Relationships, Chromosomal Location, Conserved Domains, and Gene Structure

The known protein sequences of *AtHATs* were used as query sequences, and the *B. rapa* L. protein databases were searched using “Blast Several Sequences to a Big Database” in TBtools V2.110 [68] with an e-value of 10^−5^. The whole genome sequence of *B. rapa* L. cultivar Longyou-7 was sequenced and provided by our laboratory [69]. The results of the comparison were further validated by NCBI CD-Search (https://www.ncbi.nlm.nih.gov/Structure/cdd/wrpsb.cgi, accessed on 25 March 2024). After aligning the full-length protein sequences using ClustalW2.0.10 with default parameters, MEGA 7.0 [70] was used to construct the phylogenetic tree with the maximum likelihood method. All amino acid sequences, molecular weights, isoelectric points, and hydrophobicity were predicted using the Expasy Protparam tool (https://web.expasy.org/protparam/, accessed on 9 May 2024) [71]. Using chromosome length and gene position files, the chromosomal distributions of *BrHATs* were acquired and visualized using “Gene Location Visualize (Advanced)” in TBtools V2.110 [72]. The *cis*-acting elements of HAT family members were analyzed using PLANTCARE (https://bioinformatics.psb.ugent.be/webtools/plantcare/html/, accessed on 25 March 2024) [73]. The conserved motifs of the *BrHATs* were analyzed using the online program MEME (https://meme-suite.org/meme/tools/meme, accessed on 25 March 2024) [68], and gene structures were predicted using General Feature Format (GFF3) files. Subcellular localization was predicted using the online software Plant-mPLoc (http://www.csbio.sjtu.edu.cn/bioinf/plant-multi/, accessed on 25 March 2024) [74]. Gene ontology information for the *BrHAT* genes was obtained using the eggNOG-mapper website (http://eggnog-mapper.embl.de/, accessed on 25 March 2024.). The protein sequences were analyzed and the results were visualized using TBtools software. The co-expression analysis was performed using transcriptome data, and protein interaction prediction network maps were drawn based on the co-expression results; positive correlations are shown in red, and negative correlations are shown in green.

### 4.2. Plant Materials, Growth Conditions, and Treatments

Winter rapeseed (*B. rapa* L.) cultivars CT-2360 (Caotan, Gansu), MXW-1 (Lanzhou, Gansu), 2018-FJT (Fanjiatun, Jilin), and DT-9 (Datong, Shanxi) were domesticated in different regions and have varying degrees of cold tolerance; 2018-FJT has the highest cold tolerance, followed by DT-9, MXW-1, and CT-2360 [75]. Uniform seeds were sterilized with 75% ethanol for 30 s; this was followed by soaking in 1% sodium hypochlorite solution for 5 min and washing with distilled water five times. Seedlings grown to the five-leaf stage with consistent growth were subjected to low-temperature treatment, and the leaves and roots of the four experimental materials after treatment were collected, quickly frozen in liquid nitrogen, and stored at −80 °C for future analysis. 

The seeds were then subjected to the following treatments (TSA (MedChemExpress, HY-15144, Shanghai, China), curcumin (MedChemExpress, HY-N0005, Shanghai, China) with dimethyl sulfoxide (DMSO) as solvent):

- TSA treatment

Germination test: Five treatments were established on 1/2 MS medium: CK (control), 1 μM TSA (T1), CK1 (control with DMSO equal to T1), 3 μM TSA (T2), and CK2 (control with DMSO equal to T2). Three replicates per variety were conducted at 4 °C (in a low-temperature incubator) and 24 °C (in an artificial climate incubator).

Spraying test: Nine treatments were performed in the spraying test: 4 °C, 0 °C, −4 °C, 4 °C + 50 μM TSA, 4 °C + 100 μM TSA, 0 °C+ 50 μM TSA, 0 °C+ 100 μM TSA, −4 °C + 50 μM TSA, and −4 °C + 100 μM TSA. Three biological replicates were performed for 12 h and 24 h, and leaves were collected for measurements of physiological indexes [76].

B. Curcumin treatment

Germination test: Four treatments were performed in the germination test: CK (ddH2O), 50 μM curcumin (M1), 200 μM curcumin (M2), and 350 μM curcumin (M3). Seeds were immersed for 24 h, cleaned, and sterilized, with three replicates per variety at 4 °C and 24 °C.

Spraying experiment: Four curcumin treatments were performed in the spraying experiment: 4 °C + 40 μM curcumin, 4 °C + 90 μM curcumin, −4 °C + 40 μM curcumin, and −4 °C + 90 μM curcumin. Three biological replicates of each treatment were conducted for 6 h, 12 h, and 24 h, and leaves were collected for measurements of physiological indexes. 

During seed germination, the germination potential (GE) was calculated starting on the 3rd day at 24 °C, and the germination percentage (GP) was calculated starting on the 7th day; radicle length and hypocotyl measurements were also carried out on this day. In the 4 °C treatment, GE was measured on the 8th day, and GP was assessed on the 12th day during seed germination at 4 °C. 

The calculations of GE and GP were as follows:

GE = number of germinated seeds in the first count/total number of seeds in the germination test.

GP = number of germinated seeds in the final count/total number of seeds in the germination test.

### 4.3. Analysis of Biochemical Indexes

Soluble protein content (SP) was determined by Bradford’s protocol and a standard curve was plotted to calculate the SP concentration of the samples [77]. Proline (PRO) content was determined by sulfosalicylic acid extraction followed by heat treatment with acidic ninhydrin, and finally, extracted with toluene and colourimetrically at 520 nm [78]. Malondialdehyde (MDA) content was determined by the TBARS-TCA method using 2-thiobarbituric acid (TBA, Solarbio, G8680, Beijing, China) as a substrate, which reacts with MDA to form 3,5,5-trimethyloxazole-2,4-dione under high-temperature and acidic conditions, and the difference in absorbance values was determined at two wavelengths, 532 nm and 600 nm [79]. Superoxide dismutase (SOD) activity was determined by photochemical reduction in a reaction with Nitroblue tetrazolium chloride (NBT, Solarbio, IN0110, Beijing, China), where the enzyme solution was photo-reacted with a reaction solution containing methionine, riboflavin, PBS buffer, and NBT, and the absorbance value at 560 nm was determined after 5 min [80]. Peroxidase (POD) activity was measured by the guaiacol method, where peroxidase was reacted with hydrogen peroxide in a reaction solution with guaiacol (Solarbio, SG9280, Beijing, China), and after 10 min the reaction was zeroed with a control and the absorbance value was measured at 470 nm [81]. Catalase (CAT) activity was determined by using boiled enzyme solution as a blank, H_2_O_2_ solution was added to the blank and experimental groups, and readings were taken at 240 nm at one minute intervals for a total of four readings and then calculated [82]. HAT and HDAC activities were measured using ELISA kits (sinobestbio, YX-21901P, YX-E070113P, Shanghai, China). Each index was measured in triplicate. Subsequent data analysis and plotting were performed using IBM SPSS Statistics 22.0 and Origin 2021 software.

### 4.4. Expression Pattern Analysis of BrHATs 

Previously published transcriptome data from our lab were used to analyze the expression of *BrHATs* under 4 °C treatment [83] and at different overwintering stages [5]. The transcriptome sequencing of leaves of five-leaf stage *B. rapa* L. treated at 4 °C was performed and analyzed in comparison with the control (normal growth at 22 °C). During the overwintering period, we selected the same proportion of root collar tissue (5 mm sections below the cotyledonary node) for transcriptome sequencing and compared the sequencing results with those of the pre-overwintering period (S1 stage). In this study, the overwintering (S3 and S5 stages) and rejuvenation (S6 stage) stages, which showed significant changes throughout the overwintering period, were selected for analysis. False discovery rate (FDR) > 0.001, *p* value < 0.05, and fold change > 2 or fold change < 0.5 were set as the threshold to identify significant differentially expressed genes (DEGs). Finally, the expression patterns of *BrHATs* gene family members were identified and heat-mapped in the above results. The expression of *BrHATs* after treatment with low temperature and different inhibitors was analyzed by qPCR. Five-leaf stage plants of four *B. rapa* L. grown in pots were treated in a low-temperature incubator for different periods of time and sampled at 4 °C, 0 °C, −4 °C and different inhibitor sprays, respectively; leaves and roots were collected from the low-temperature-treated material, only the leaves were collected from the inhibitor-treated material, and normal-growing plants at 22 °C were used as the calibration sample for the four varieties. Total RNA was extracted using the Plant RNA Extraction Kit (TIANGEN, DP431, Beijing, China), and reverse transcription was performed using the Reverse Transcription Kit (TIANGEN, KR118-02, Beijing, China). The real-time fluorescence quantitative PCR analysis of *HAT* family genes was conducted using synthesized cDNA as a template (TIANGEN, Super Real premix Plus (SYBR Green) (FP205), Beijing, China). The PCR program consisted of 40 cycles at 95 °C for 15 min, 95 °C for 10 s, and 60 °C for 30 s. *Actin* was used as the internal reference gene, and the data were processed using the 2^−ΔΔCT^ method [84]. The primer sequences used are shown in Table S1, and three replications were performed for each sample. Heat maps were plotted using TBtools to display the expression levels obtained from the different tissues of *B. rapa* L. after different treatments.

## 5. Conclusions

A total of 29 *BrHAT* genes were identified, and these were distributed across 10 chromosomes and classified into four subfamilies. We analyzed the sequences and promoters of *BrHATs*. The *cis*-acting element analysis of the promoter sequences of *BrHATs* revealed conserved and species-specific *cis*-acting elements related to responses to light, hormones, and stresses, as well as growth and development. GO enrichment analysis and protein prediction further suggest that these genes play different roles in the resistance process. The results of the HAT and HDAC inhibitor treatments showed that these two inhibitors not only regulate the dynamic balance of histone acetylation–deacetylation, but also play a role in the low-temperature response during seed germination and regulate the activity of peroxidase and the accumulation of soluble substances in the plant body under low-temperature stress, which improves the low-temperature resistance of plants. Expression analyses showed that *BrHATs* play a role in different overwintering stages and mediate responses to low temperatures; these findings provide new insights into the role of *BrHATs* in plant stress responses. Our study sheds new light on the physiological regulatory mechanisms of *BrHATs* in response to low-temperature stress and enhances our knowledge of plant *HAT* genes. Additional studies on the three differentially expressed genes (*BrHAC13*, *BrHAM1*, and *BrTAF1*) identified in our study are needed.

## Figures and Tables

**Table 1:** The physicochemical properties of *BrHAT* genes in winter rapeseed.

Gene Name	Sequence ID	Number of Amino Acid	Molecular Weight/kDa	pI	Instability Index	Hydrophilia	Aliphatic Index
*BrHAC1*	*Brapa01T000131.1*	374	42,686.6	9.08	55.25	84.41	−0.336
*BrTAF1*	*Brapa01T003430.1*	1772	201,196.61	5.69	54.09	70.56	−0.799
*BrTAF2*	*Brapa01T003498.1*	305	34,158.23	6.94	28.92	102.26	−0.429
*BrHAC9*	*Brapa01T003886.1*	1637	184,450.74	8.85	50.11	66.76	−0.671
*BrHAC10*	*Brapa02T001822.1*	1012	116,033	8.01	44.88	70.3	−0.565
*BrHAC11*	*Brapa02T002581.1*	1653	185,472.72	8.71	57.08	62.03	−0.737
*BrHAC2*	*Brapa02T004562.1*	368	41,825.9	9.45	64.14	87.39	−0.313
*BrTAF3*	*Brapa03T002862.1*	305	34,192.25	6.94	29.2	100.98	−0.434
*BrHAC12*	*Brapa03T003605.1*	1603	181,371.88	8.81	52.63	65.31	−0.679
*BrHAC3*	*Brapa03T006148.1*	379	43,316.25	8.92	54.44	83.83	−0.405
*BrHAG1*	*Brapa04T000545.1*	525	59,015.22	6.09	41.82	70.1	−0.62
*BrHAG2*	*Brapa04T000549.1*	548	60,926.09	6.13	40.5	69.29	−0.624
*BrTAF4*	*Brapa05T002439.1*	229	25,685.49	6.86	29.17	100.87	−0.431
*BrHAC8*	*Brapa06T001216.1*	990	110,352.88	6.71	51.87	69.22	−0.598
*BrHAM1*	*Brapa06T002435.1*	160	18,855.76	6.17	48.73	79.25	−0.456
*BrTAF5*	*Brapa06T002581.1*	381	42,699.02	7	29.22	101.05	−0.436
*BrHAC4*	*Brapa06T002586.1*	380	43,636.84	9.16	44.78	82.84	−0.357
*BrHAC13*	*Brapa07T003346.1*	876	100,268.81	6.36	57.76	75.21	−0.509
*BrTAF6*	*Brapa08T000884.1*	1893	213,267.71	5.35	52.28	72.09	−0.816
*BrTAF7*	*Brapa08T001640.1*	229	25,685.49	6.86	29.17	100.87	−0.431
*BrHAC14*	*Brapa08T003011.1*	1578	176,696.25	8.74	53.39	64.27	−0.724
*BrHAC5*	*Brapa08T003012.1*	297	32,073.09	6.76	57.55	75.52	−0.525
*BrHAC6*	*Brapa08T003480.1*	369	41,612.29	9.3	52.74	81.36	−0.341
*BrHAC15*	*Brapa09T000822.1*	1653	183,826.65	8.72	50.79	65.36	−0.667
*BrTAF8*	*Brapa09T004162.1*	229	25,685.49	6.86	29.17	100.87	−0.431
*BrHAM2*	*Brapa09T005768.1*	465	54,007.8	6.81	44.2	83.2	−0.515
*BrHAM3*	*Brapa10T000566.1*	441	50,864.11	7.56	40.61	78.25	−0.619
*BrHAC15*	*Brapa10T001880.1*	1712	192,652.85	5.58	42.45	90.95	−0.21
*BrHAC7*	*Brapa10T002727.1*	955	106,960.73	8.11	46.98	89.75	−0.155

## Data Availability

The data presented in this study are available in this article and the Supplementary Materials.

## Supplementary Materials

The following supporting information can be downloaded at: https://www.mdpi.com/article/10.3390/ijms25179200/s1.

## References

[1] Ma L., Wang X., Pu Y., Wu J., Coulter J.A., Li X., Wang L., Liu L., Fang Y., Niu Z. (2019). Ecological and economic benefits of planting winter rapeseed (*Brassica rapa* L.) in the wind erosion area of northern China. Sci. Rep..

[2] Bewick A.J., Ji L., Niederhuth C.E., Willing E.-M., Hofmeister B.T., Shi X., Wang L., Lu Z., Rohr N.A., Hartwig B. (2016). On the origin and evolutionary consequences of gene body DNA methylation. Proc. Natl. Acad. Sci. USA.

[3] Chang Y.N., Zhu C., Jiang J., Zhang H., Zhu J.K., Duan C.G. (2020). Epigenetic regulation in plant abiotic stress responses. J. Integr. Plant Biol..

[4] Xing G., Jin M., Qu R., Zhang J., Han Y., Han Y., Wang X., Li X., Ma F., Zhao X. (2022). Genome-wide investigation of histone acetyltransferase gene family and its responses to biotic and abiotic stress in foxtail millet (*Setaria italica* [L.] P. Beauv). BMC Plant Biol..

[5] Liu L., Pu Y., Niu Z., Wu J., Fang Y., Xu J., Xu F., Yue J., Ma L., Li X. (2022). Transcriptomic Insights Into Root Development and Overwintering Transcriptional Memory of *Brassica rapa* L. Grown in the Field. Front. Plant Sci..

[6] Zhu M., Monroe J.G., Suhail Y., Villiers F., Mullen J., Pater D., Hauser F., Jeon B.W., Bader J.S., Kwak J.M. (2016). Molecular and systems approaches towards drought-tolerant canola crops. New Phytol..

[7] Wang J., Jiao J., Zhou M., Jin Z., Yu Y., Liang M. (2019). Physiological and Transcriptional Responses of Industrial Rapeseed (*Brassica napus*) Seedlings to Drought and Salinity Stress. Int. J. Mol. Sci..

[8] Pan J., Zhang L., Chen M., Ruan Y., Li P., Guo Z., Liu B., Ruan Y., Xiao M., Huang Y. (2022). Identification and charactering of APX genes provide new insights in abiotic stresses response in *Brassica napus*. PeerJ.

[9] Ali I., Conrad R.J., Verdin E., Ott M. (2018). Lysine Acetylation Goes Global: From Epigenetics to Metabolism and Therapeutics. Chem. Rev..

[10] Zhang X., Wan Q., Liu F., Zhang K., Sun A., Luo B., Sun L., Wan Y. (2015). Molecular analysis of the chloroplast Cu/Zn-SOD gene (AhCSD2) in peanut. Crop J..

[11] Wu C.-L., Lin L.-F., Hsu H.-C., Huang L.-F., Hsiao C.-D., Chou M.-L. (2021). *Saussurea involucrata* (Snow Lotus) *ICE1* and *ICE2* Orthologues Involved in Regulating Cold Stress Tolerance in Transgenic *Arabidopsis*. Int. J. Mol. Sci..

[12] Hwarari D., Guan Y., Ahmad B., Movahedi A., Min T., Hao Z., Lu Y., Chen J., Yang L. (2022). ICE-CBF-COR Signaling Cascade and Its Regulation in Plants Responding to Cold Stress. Int. J. Mol. Sci..

[13] Cui K., Xu L., Tao T., Huang L., Li J., Hong J., Li H., Chi Y. (2023). Mechanical behavior of multiscale hybrid fiber reinforced recycled aggregate concrete subject to uniaxial compression. J. Build. Eng..

[14] Kong L., Liu Y., Wang X., Chang C. (2020). Insight into the Role of Epigenetic Processes in Abiotic and Biotic Stress Response in Wheat and Barley. Int. J. Mol. Sci..

[15] Pandey R., Muller A., Napoli C.A., Selinger D.A., Pikaard C.S., Richards E.J., Bender J., Mount D.W., Jorgensen R.A. (2002). Analysis of histone acetyltransferase and histone deacetylase families of Arabidopsis thaliana suggests functional diversification of chromatin modification among multicellular eukaryotes. Nucleic Acids Res..

[16] Yuan L., Liu X., Luo M., Yang S., Wu K. (2013). Involvement of histone modifications in plant abiotic stress responses. J. Integr. Plant Biol..

[17] Zhu X., Li Q., Chang R., Yang D., Song Z., Guo Q., Huang C. (2014). Curcumin Alleviates Neuropathic Pain by Inhibiting p300/CBP Histone Acetyltransferase Activity-Regulated Expression of BDNF and Cox-2 in a Rat Model. PLoS ONE.

[18] Balasubramanyam K., Varier R.A., Altaf M., Swaminathan V., Siddappa N.B., Ranga U., Kundu T.K. (2004). Curcumin, a Novel p300/CREB-binding Protein-specific Inhibitor of Acetyltransferase, Represses the Acetylation of Histone/Nonhistone Proteins and Histone Acetyltransferase-dependent Chromatin Transcription. J. Biol. Chem..

[19] Ailenberg M., Silverman M. (2002). Trichostatin A—Histone deacetylase inhibitor with clinical therapeutic potential—Is also a selective and potent inhibitor of gelatinase A expression. Biochem. Biophys. Res. Commun..

[20] Deng Z., Liu X., Jin J., Xu H., Gao Q., Wang Y., Zhao J. (2016). Histone Deacetylase Inhibitor Trichostatin a Promotes the Apoptosis of Osteosarcoma Cells through p53 Signaling Pathway Activation. Int. J. Biol. Sci..

[21] Hu Y., Lu Y., Zhao Y., Zhou D.-X. (2019). Histone Acetylation Dynamics Integrates Metabolic Activity to Regulate Plant Response to Stress. Front. Plant Sci..

[22] Kölle D., Sarg B., Lindner H., Loidl P. (1998). Substrate and sequential site specificity of cytoplasmic histone acetyltransferases of maize and rat liver. FEBS Lett..

[23] Lusser A., Eberharter A., Loidl A., Schramel M.G., Horngacher M., Haas H., Loidl P. (1999). Analysis of the histone acetyltransferase B complex of maize embryos. Nucleic Acids Res..

[24] Marmorstein R. (2001). Structure and function of histone acetyltransferases. Cell. Mol. Life Sci..

[25] Latham J.A., Dent S.Y.R. (2007). Cross-regulation of histone modifications. Nat. Struct. Mol. Biol..

[26] Allfrey V.G., Faulkner R., Mirsky A.E. (1964). Acetylation and methylation of histones and their possible role in the regulation of rna synthesis. Proc. Natl. Acad. Sci. USA.

[27] Wei J., Zheng G., Yu X., Liu S., Dong X., Cao X., Fang X., Li H., Jin J., Mi W. (2021). Comparative Transcriptomics and Proteomics Analyses of Leaves Reveals a Freezing Stress-Responsive Molecular Network in Winter Rapeseed (*Brassica rapa* L.). Front. Plant Sci..

[28] Pu Y., Liu L., Wu J., Zhao Y., Bai J., Ma L., Yue J., Jin J., Niu Z., Fang Y. (2019). Transcriptome Profile Analysis of Winter Rapeseed (*Brassica napus* L.) in Response to Freezing Stress, Reveal Potentially Connected Events to Freezing Stress. Int. J. Mol. Sci..

[29] Zeng X., Xu Y., Jiang J., Zhang F., Ma L., Wu D., Wang Y., Sun W. (2018). iTRAQ-Based Comparative Proteomic Analysis of the Roots of TWO Winter Turnip Rapes (*Brassica rapa* L.) with Different Freezing-Tolerance. Int. J. Mol. Sci..

[30] Wang J., Chitsaz F., Derbyshire M.K., Gonzales N.R., Gwadz M., Lu S., Marchler G.H., Song J.S., Thanki N., Yamashita R.A. (2022). The conserved domain database in 2023. Nucleic Acids Res..

[31] Gamsjaeger R., Liew C., Loughlin F., Crossley M., Mackay J. (2007). Sticky fingers: Zinc-fingers as protein-recognition motifs. Trends Biochem. Sci..

[32] Jayaram H.N., Cooney D.A., A Ryan J., Neil G., Dion R.L., Bono V.H. (1975). L-[alphaS, 5S]-alpha-amino-3-chloro-4,5-dihydro-5-isoxazoleacetic acid (NSC-163501): A new amino acid antibiotic with the properties of an antagonist of L-glutamine. Cancer Chemother. Rep..

[33] Latrasse D., Benhamed M., Henry Y., Domenichini S., Kim W., Zhou D.-X., Delarue M. (2008). The MYST histone acetyltransferases are essential for gametophyte development in Arabidopsis. BMC Plant Biol..

[34] Shamshad A., Rashid M., Zaman Q.U. (2023). In-silico analysis of heat shock transcription factor (OsHSF) gene family in rice (*Oryza sativa* L.). BMC Plant Biol..

[35] Hu W., Hou X., Huang C., Yan Y., Tie W., Ding Z., Wei Y., Liu J., Miao H., Lu Z. (2015). Genome-Wide Identification and Expression Analyses of Aquaporin Gene Family during Development and Abiotic Stress in Banana. Int. J. Mol. Sci..

[36] Sun J., Tian Z., Li X., Li S., Li Z., Wang J., Hu Z., Chen H., Guo C., Xie M. (2022). Systematic analysis of the pectin methylesterase gene family in Nicotiana tabacum and reveal their multiple roles in plant development and abiotic stresses. Front. Plant Sci..

[37] Zhao Z., Zhu J., Li J., Li Z., Li Y. (2023). Genome-wide Identification and Expressions under Stresses of RLCK VI Family in Gossypium barbadense. Fujian Agric. J..

[38] Pfaffl M.W. (2001). A new mathematical model for relative quantification in real-time RT-PCR. Nucleic Acids Res..

[39] Huo C., Zhang B., Wang R. (2021). Research progress on plant noncoding RNAs in response to low-temperature stress. Plant Signal. Behav..

[40] Ueda M., Seki M. (2019). Histone Modifications Form Epigenetic Regulatory Networks to Regulate Abiotic Stress Response. Plant Physiol..

[41] Kuo M.H., Allis C.D. (1998). Roles of histone acetyltransferases and deacetylases in gene regulation. Bioessays.

[42] Liu X., Luo M., Zhang W., Zhao J., Zhang J., Wu K., Tian L., Duan J. (2012). Histone acetyltransferases in rice (*Oryza sativa* L.): Phylogenetic analysis, subcellular localization and expression. BMC Plant Biol..

[43] Kubo T., Yoshimura A., Kurata N. (2022). Loss of OsEAF6, a Subunit of the Histone Acetyltransferase Complex, Causes Hybrid Breakdown in Intersubspecific Rice Crosses. Front. Plant Sci..

[44] Han Y., Georgii E., Priego-Cubero S., Wurm C.J., Hüther P., Huber G., Koller R., Becker C., Durner J., Lindermayr C. (2023). Arabidopsis histone deacetylase HD2A and HD2B regulate seed dormancy by repressing DELAY OF GERMINATION 1. Front. Plant Sci..

[45] Poulios S., Tsilimigka F., Mallioura A., Pappas D., Seira E., Vlachonasios K. (2022). Histone Acetyltransferase GCN5 Affects Auxin Transport during Root Growth by Modulating Histone Acetylation and Gene Expression of PINs. Plants.

[46] Hendrickson W.A., Ward K.B. (1975). Atomic models for the polypeptide backbones of myohemerythrin and hemerythrin. Biochem. Biophys. Res. Commun..

[47] Dincer I., Temiz M. (2024). Energy, Environment, and Sustainable Development. Renewable Energy Options for Power Generation and Desalination.

[48] Huang M., Huang J., Zheng Y., Sun Q. (2019). Histone acetyltransferase inhibitors: An overview in synthesis, structure-activity relationship and molecular mechanism. Eur. J. Med. Chem..

[49] Hulse-Kemp A.M., Bostan H., Chen S., Ashrafi H., Stoffel K., Sanseverino W., Li L., Cheng S., Schatz M.C., Garvin T. (2021). An anchored chromosome-scale genome assembly of spinach improves annotation and reveals extensive gene rearrangements in euasterids. Plant Genome.

[50] Guo M., Wang S., Liu H., Yao S., Yan J., Wang C., Miao B., Guo J., Ma F., Guan Q. (2023). Histone deacetylase MdHDA6 is an antagonist in regulation of transcription factor MdTCP15 to promote cold tolerance in apple. Plant Biotechnol. J..

[51] Xu C.-R., Liu C., Wang Y.-L., Li L.-C., Chen W.-Q., Xu Z.-H., Bai S.-N. (2005). Histone acetylation affects expression of cellular patterning genes in the Arabidopsis root epidermis. Proc. Natl. Acad. Sci. USA.

[52] Michaud P.-A. (2010). Adolescent medicine in Guglera: A novel approach for unemployed, severely obese adolescents. Rev. Medicale Suisse.

[53] Nelson S.K., Ariizumi T., Steber C.M. (2017). Biology in the Dry Seed: Transcriptome Changes Associated with Dry Seed Dormancy and Dormancy Loss in the Arabidopsis GA-Insensitive sleepy1-2 Mutant. Front. Plant Sci..

[54] Zhang X., Wang K., Ervin E., Waltz C., Murphy T. (2011). Metabolic Changes During Cold Acclimation and Deacclimation in Five Bermudagrass Varieties. I. Proline, Total Amino Acid, Protein, and Dehydrin Expression. Crop Sci..

[55] Kefu Z., Hai F., Ungar I. (2002). Survey of halophyte species in China. Plant Sci..

[56] Wang L., Yuan J., Ma Y., Jiao W., Ye W., Yang D.-L., Yi C., Chen Z.J. (2017). Rice Interploidy Crosses Disrupt Epigenetic Regulation, Gene Expression, and Seed Development. Mol. Plant.

[57] Bose K.S., Sarma R.H. (1975). Delineation of the intimate details of the backbone conformation of pyridine nucleotide coenzymes in aqueous solution. Biochem. Biophys. Res. Commun..

[58] Liu H., Luo J., Fang L., Huang H., Deng J., Huang J., Zhang S., Li Y., Zheng J. (2018). An electrochemical strategy with tetrahedron rolling circle amplification for ultrasensitive detection of DNA methylation. Biosens. Bioelectron..

[59] Yu Y., Cao H., Zhang M., Shi F., Wang R., Liu X. (2018). Prognostic value of DNA methylation for bladder cancer. Clin. Chim. Acta.

[60] Santos H.P., Nephew B.C., Bhattacharya A., Tan X., Smith L., Alyamani R.A.S., Martin E.M., Perreira K., Fry R.C., Murgatroyd C. (2018). Discrimination exposure and DNA methylation of stress-related genes in Latina mothers. Psychoneuroendocrinology.

[61] Tulloch J., Leong L., Chen S., Keene C.D., Millard S.P., Shutes-David A., Lopez O.L., Kofler J., Kaye J.A., Woltjer R. (2018). *APOE* DNA methylation is altered in Lewy body dementia. Alzheimer’s Dement..

[62] El Baidouri M., Kim K.D., Abernathy B., Li Y.-H., Qiu L.-J., Jackson S.A. (2018). Genic C-Methylation in Soybean Is Associated with Gene Paralogs Relocated to Transposable Element-Rich Pericentromeres. Mol. Plant.

[63] Huminiecki L., Horbańczuk J., Atanasov A.G. (2017). The functional genomic studies of curcumin. Semin. Cancer Biol..

[64] Mohajeri M., Sahebkar A. (2018). Protective effects of curcumin against doxorubicin-induced toxicity and resistance: A review. Crit. Rev. Oncol..

[65] Semik-Gurgul E., Ząbek T., Fornal A., Wnuk M., Pawlina-Tyszko K., Gurgul A., Klukowska-Rötzler J., Koch C., Mählmann K., Bugno-Poniewierska M. (2018). DNA methylation patterns of the S100A14, POU2F3 and SFN genes in equine sarcoid tissues. Res. Vet. Sci..

[66] Ak T., Gülçin I. (2008). Antioxidant and radical scavenging properties of curcumin. Chem. Interact..

[67] Bouyahya A., El Omari N., Bakha M., Aanniz T., El Menyiy N., El Hachlafi N., El Baaboua A., El-Shazly M., Alshahrani M.M., Al Awadh A.A. (2022). Pharmacological Properties of Trichostatin A, Focusing on the Anticancer Potential: A Comprehensive Review. Pharmaceuticals.

[68] Bailey T.L., Johnson J., Grant C.E., Noble W.S. (2015). The MEME Suite. Nucleic Acids Res..

[69] Wu J., Xu X.-D., Liu L., Ma L., Pu Y., Wang W., Hua X.-Y., Song J.-M., Liu K., Lu G. (2022). A Chromosome Level Genome Assembly of a Winter Turnip Rape (*Brassica rapa* L.) to Explore the Genetic Basis of Cold Tolerance. Front. Plant Sci..

[70] Hall B.G. (2013). Building Phylogenetic Trees from Molecular Data with MEGA. Mol. Biol. Evol..

[71] Wilkins M.R., Gasteiger E., Bairoch A., Sanchez J.C., Williams K.L., Appel R.D., Hochstrasser D.F. (1999). Protein Identification and Analysis Tools in the ExPASy Server. 2-D Proteome Analysis Protocols.

[72] Chen C.J., Chen H., Zhang Y., Thomas H.R., Frank M.H., He Y.H., Xia R. (2020). TBtools: An Integrative Toolkit Developed for Interactive Analyses of Big Biological Data. Mol. Plant.

[73] Lescot M., Déhais P., Thijs G., Marchal K., Moreau Y., Van de Peer Y., Rouzé P., Rombauts S. (2002). PlantCARE, a database of plant cis-acting regulatory elements and a portal to tools for in silico analysis of promoter sequences. Nucleic Acids Res..

[74] Chou K.C., Shen H.B. (2010). Plant-Mploc: A top-down strategy to augment the power for predicting plant protein dubcellular localization. PLoS ONE.

[75] Wang W.P., Ma L., Sun B.L., Guo X.J., Wang S.F., Niu Z.X., Qi W.L., Pu Y.Y., Lu X.M., Hu F.D. (2021). Physiological mechanism of DNA demethylation in improving the cold resistance of *Brassica rapa* L.. Agric. Res. Arid. Areas.

[76] Xu F., Liu L., Li P., Yao Y., Sun W., Wu J. (2022). Identification and Expression Analysis of HDACs Gene Family in Winter Rapeseed (*Brassica rapa* L.). Acta Bot. Boreali-Occident. Sin..

[77] Gasparov V.S., Degtiar V.G. (1994). Opredelenie belka po sviazyvaniiu s krasitelem kumassi brilliantovym golubym G-250 [Protein determination by binding with the dye Coomassie brilliant blue G-250]. Biokhimiia.

[78] Bor M., Özdemir F., Türkan I. (2003). The effect of salt stress on lipid peroxidation and antioxidants in leaves of sugar beet *Beta vulgaris* L. and wild beet *Beta maritima* L.. Plant Sci..

[79] Demiral T., Türkan I. (2005). Comparative lipid peroxidation, antioxidant defense systems and proline content in roots of two rice cultivars differing in salt tolerance. Environ. Exp. Bot..

[80] Spitz D.R., Oberley L.W. (1989). An assay for superoxide dismutase activity in mammalian tissue homogenates. Anal. Biochem..

[81] Doerge D.R., Divi R.L., Churchwell M.I. (1997). Identification of the Colored Guaiacol Oxidation Product Produced by Peroxidases. Anal. Biochem..

[82] He H., Lei Y., Yi Z., Raza A., Zeng L., Yan L., Xiaoyu D., Yong C., Xiling Z. (2021). Study on the mechanism of exogenous serotonin improving cold tolerance of rapeseed (*Brassica napus* L.) seedlings. Plant Growth Regul..

[83] Ma L., Coulter J.A., Liu L., Zhao Y., Chang Y., Pu Y., Zeng X., Xu Y., Wu J., Fang Y. (2019). Transcriptome Analysis Reveals Key Cold-Stress-Responsive genes in Winter Rapeseed (*Brassica rapa* L.). Int. J. Mol. Sci..

[84] Rao X., Huang X., Zhou Z., Lin X. (2013). An improvement of the 2^−ΔΔCT^ method for quantitative real-time polymerase chain reaction data analysis. Biostat. Bioinform. Biomath..

