# Supplementary material for: Systematic Analysis of the BrHAT Gene Family and Physiological Characteristics of Brassica rapa L. Treated with Histone Acetylase and Deacetylase Inhibitors under Low Temperature
Source: Int J Mol Sci. 2024 Aug 24;25(17):9200. doi: 10.3390/ijms25179200 (PMC11395008; doi:10.3390/ijms25179200)
Supplement: Supplementary file 1 [file ijms-25-09200-s001.zip › Supplementary figures.pdf]

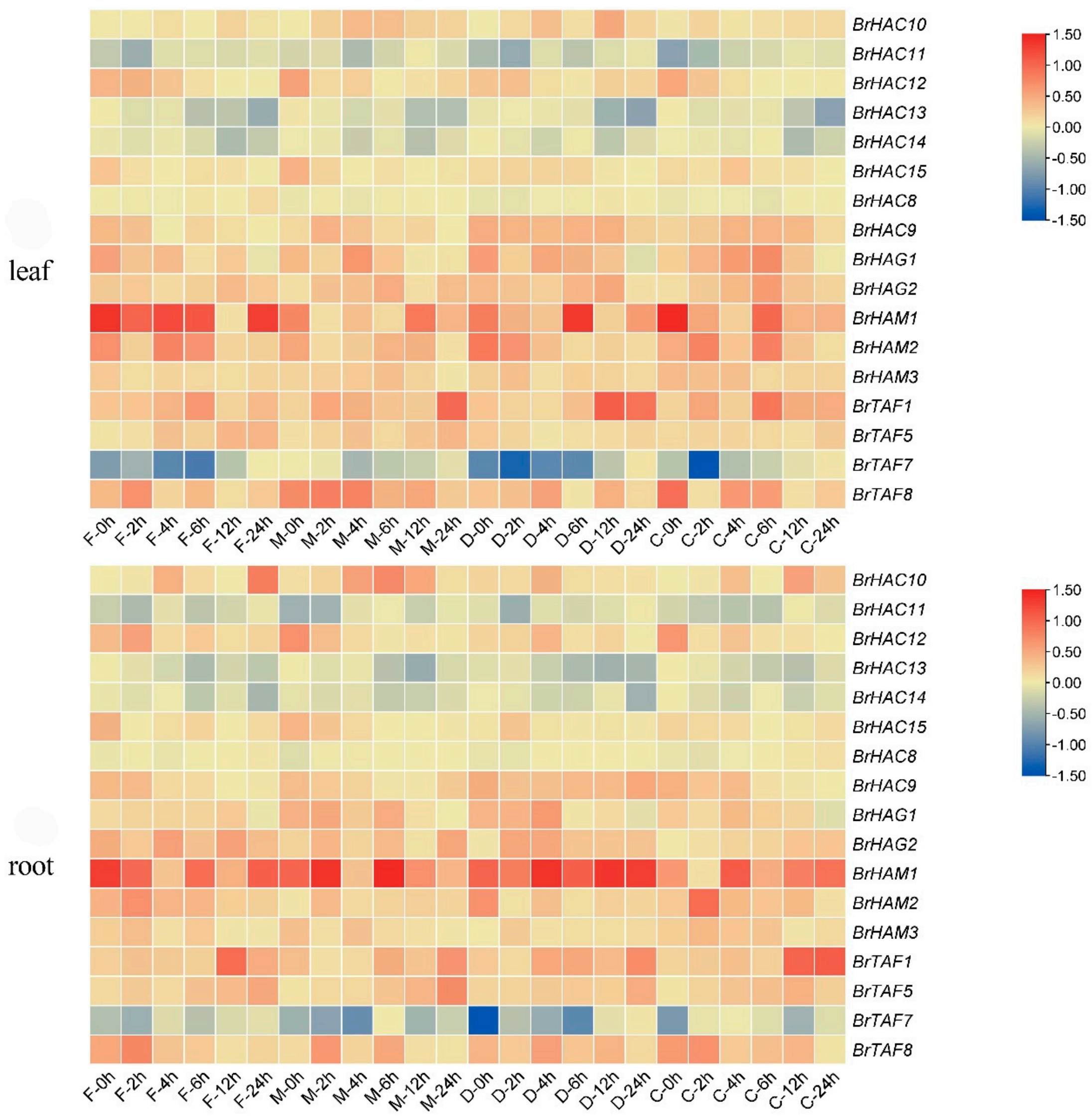

Figure S1. Expression analysis of BrHAT family members in leaves and roots under 0℃. “C” is “CT-2360”, “M” is “MXW-1”, “D” is” DT-9”, “F” is “2018-FJT”.

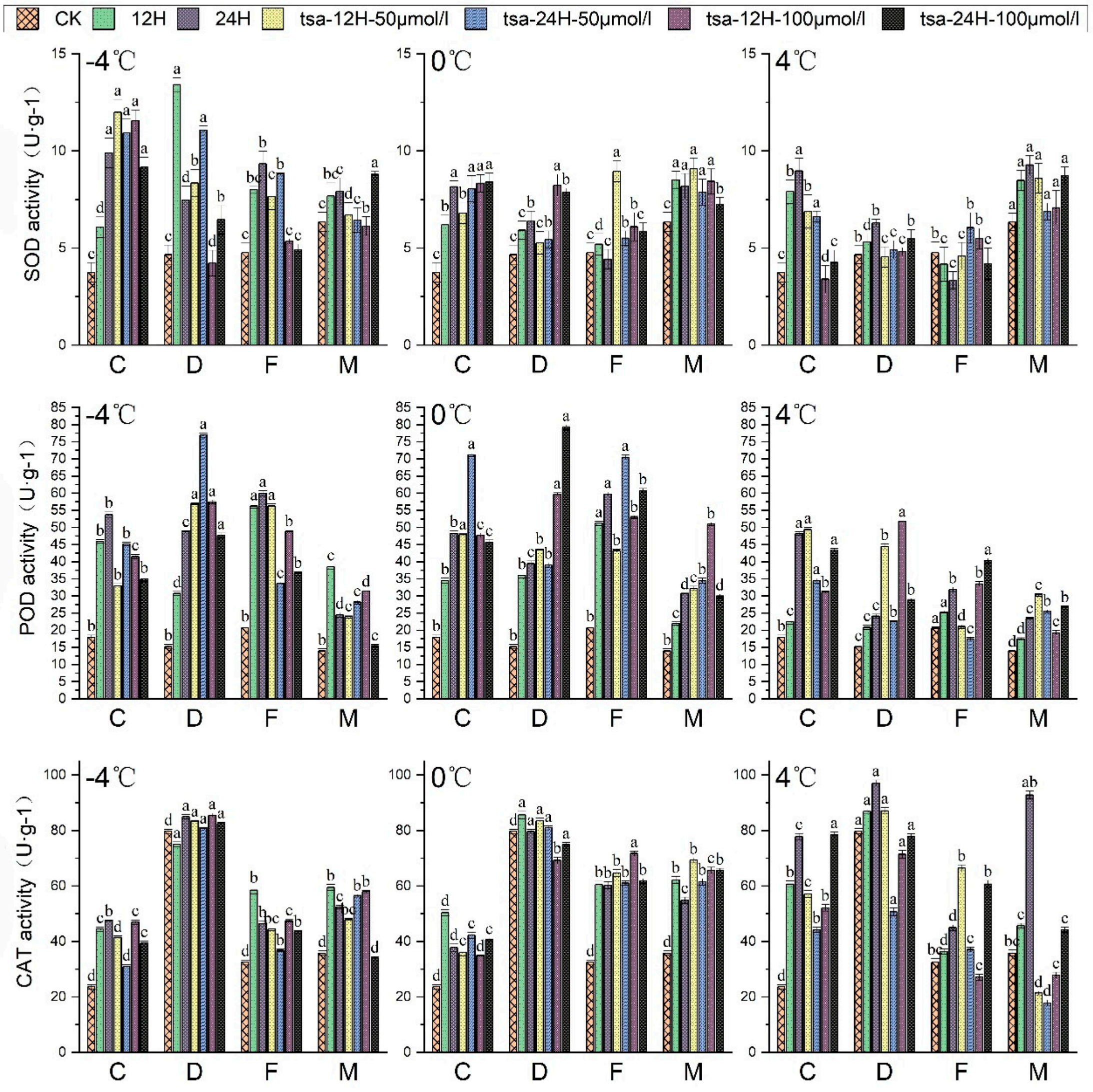

Figure S2. Antioxidant enzyme activity under different TSA concentrations. “C” is “CT-2360”, “M” is “MXW-1”, “D” is” DT-9”, “F” is “2018-FJT”. Different normal letters indicate significant differences within different treatments for same variety at 0.05 level.

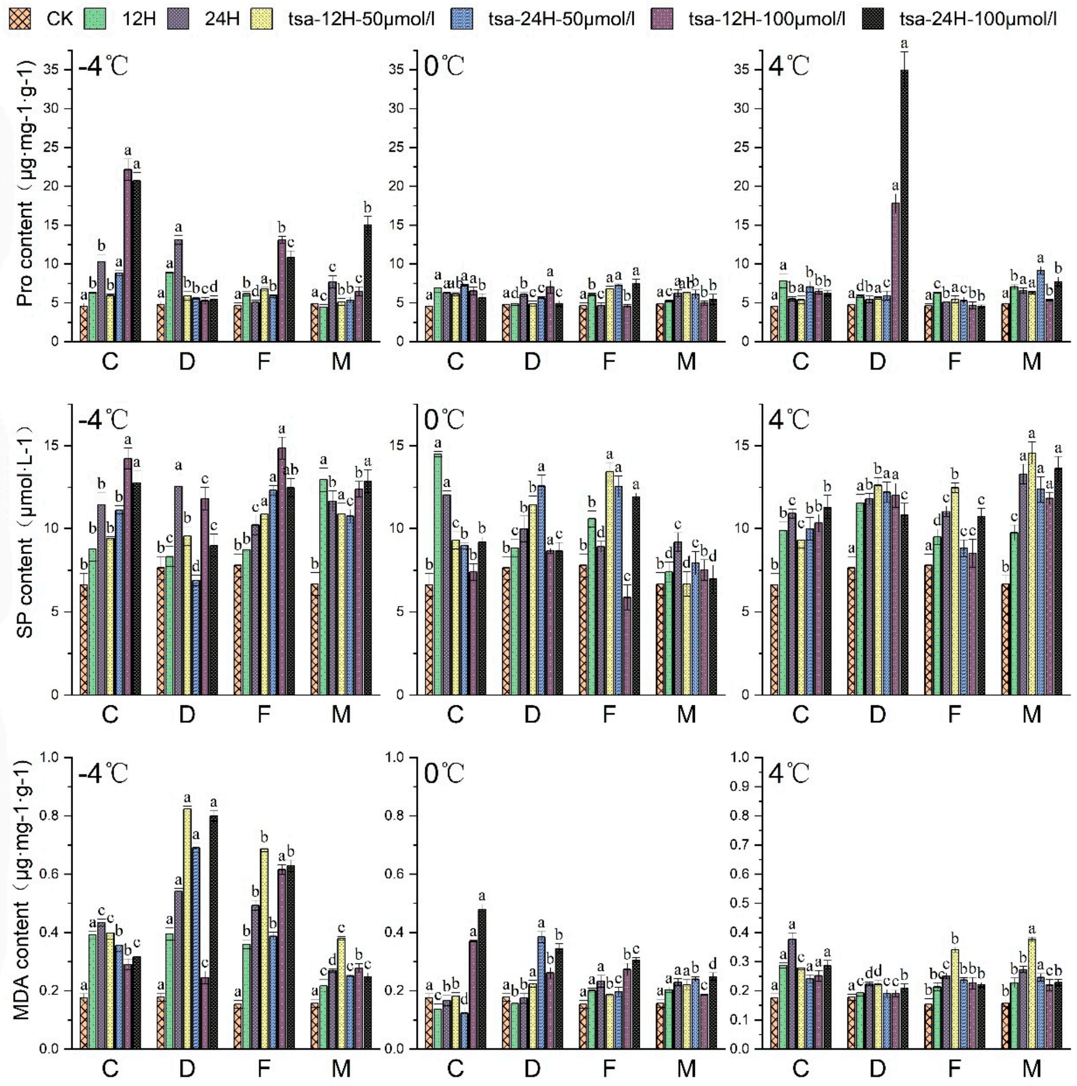

Figure S3. The contents of osmotic adjustment substances under different curcumin concentrations. “C” is “CT-2360”, “M” is “MXW-1”, “D” is” DT-9”, “F” is “2018-FJT”. Different normal letters indicate significant differences within different treatments for same variety at 0.05 level.
