# Supplementary material for: Systematic Analysis of the BrHAT Gene Family and Physiological Characteristics of Brassica rapa L. Treated with Histone Acetylase and Deacetylase Inhibitors under Low Temperature
Source: Int J Mol Sci. 2024 Aug 24;25(17):9200. doi: 10.3390/ijms25179200 (PMC11395008; doi:10.3390/ijms25179200)
Supplement: Supplementary file 1 [file ijms-25-09200-s001.zip › Supplementary table-Table .pdf]

Table S1 Primer sequence used in study

Gene name	FORWARD (5' to 3')	REVERSE (5' to 3')
BrHAC10	CCGTCTAAGCGACAGAGAGC	TGGCGTTCTGGGTGTATTGA
BrHAC11	CGACGAACTTTGAACCCAGAA	AGGAGCAATCCTTCGCAAGT
BrHAC12	TCAAGTAGATCCATCCAAGCCG	GTGGTATCTGCTGTGTCTGGT
BrHAC13	TGCTGTCCATCTTACCGAGC	GTCTGCTTGCTACTCACCACT
BrHAC14	GTGTTAAGAGCTGTAGTGAGATCAG	AGACTGCCCAGATCGTTGTT
BrHAC15	AATCTCTTCCTGGCTCTTCGC	ACTCTTCCACCCCAGAGAGAA
BrHAC8	CTGGGACCTCCGGTTGATTC	TGATTTCTTCCTTTTTGCGACCT
BrHAC9	CAGCGCAAACTCATCAGACG	AAGGTGAATGCCACTGTCCC
BrHAG1	CCAATCGTTCTCGCAGCTCTCAG	AGGTCGTCGTTGGAGGAAGGAG
BrHAG2	TCTTCGTCGTCGTCGTCTCCTC	TTGGCTGCGTTGAGGTGTGAAG
BrHAM1	GGGACGCCACATGGTTGGATAC	TCCGAGTCCTGTAACCTGGATGG
BrHAM2	GCATCCTTACCCTTCCGCCATATC	GGTGTCCCGACTTTTCCCTCTTTC
BrHAM3	GCGGCGATGCGTCTCTGAATC	CACAGCACACGAGTACCCACTTC
BrTAF1	CCGTCTCTTCCAATCGCTCAATCG	CCTCGTTGCGTTCAGCCATCTC
BrTAF5	GCAAGCAGTTGGAGGATGGAAGG	CGTAGACGCAAGACCAAGTGGAG
BrTAF7	GGTGTTGCGTCTGCGTGGAG	TTGTCAATGGTGTCGGAACTCTCG
BrTAF8	CTCTCCACTTGGTGTTGCGTCTAC	ACGTTGTCAATGGTGTCGGAACTC
BrHAC10	CCGTCTAAGCGACAGAGAGC	TGGCGTTCTGGGTGTATTGA
BrHAC11	CGACGAACTTTGAACCCAGAA	AGGAGCAATCCTTCGCAAGT
BrHAC12	TCAAGTAGATCCATCCAAGCCG	GTGGTATCTGCTGTGTCTGGT
